# Spinal cord from body donors is suitable for multicolor immunofluorescence

**DOI:** 10.1007/s00418-022-02154-5

**Published:** 2022-10-06

**Authors:** Lukas F. Reissig, Genova Carrero-Rojas, Udo Maierhofer, Atieh Seyedian Moghaddam, Andreas Hainfellner, Bernhard Gesslbauer, Thomas Haider, Johannes Streicher, Oskar C. Aszmann, Angel M. Pastor, Wolfgang J. Weninger, Roland Blumer

**Affiliations:** 1 Division of Anatomy, MIC, Medical University Vienna, Vienna, Austria; 2 Clinical Laboratory for Bionic Extremity Reconstruction, Department of Plastic, Reconstructive and Aesthetic Surgery, Medical University of Vienna, Vienna, Austria; 3 Department of Orthopedic and Trauma Surgery, Medical University of Vienna, Vienna, Austria; 4 Department of Anatomy and Biomechanics, Division of Anatomy and Developmental Biology, Karl Landsteiner University of Health Science, Krems an der Donau, Austria; 5 Departamento de Fisiología, Facultad de Biología, Universidad de Sevilla, Seville, Spain

**Keywords:** Human spinal cord, *Postmortem* interval, Multicolor immunofluorescence, Horseradish peroxidase immunohistochemistry, Neuronal markers

## Abstract

Immunohistochemistry is a powerful tool for studying neuronal tissue from humans at the molecular level. Obtaining fresh neuronal tissue from human organ donors is difficult and sometimes impossible. In anatomical body donations, neuronal tissue is dedicated to research purposes and because of its easier availability, it may be an alternative source for research. In this study, we harvested spinal cord from a single organ donor 2 h (h) *postmortem* and spinal cord from body donors 24, 48, and 72 h *postmortem* and tested how long after death, valid multi-color immunofluorescence or horseradish peroxidase (HRP) immunohistochemistry is possible. We used general and specific neuronal markers and glial markers for immunolabeling experiments. Here we showed that it is possible to visualize molecularly different neuronal elements with high precision in the body donor spinal cord 24 h *postmortem* and the quality of the image data was comparable to those from the fresh organ donor spinal cord. High-contrast multicolor images of the 24-h spinal cords allowed accurate automated quantification of different neuronal elements in the same sample. Although there was antibody-specific signal reduction over *postmortem* intervals, the signal quality for most antibodies was acceptable at 48 h but no longer at 72 h *postmortem.* In conclusion, our study has defined a *postmortem* time window of more than 24 h during which valid immunohistochemical information can be obtained from the body donor spinal cord. Due to the easier availability, neuronal tissue from body donors is an alternative source for basic and clinical research.

**Supplementary Information:**

The online version contains supplementary material available at 10.1007/s00418-022-02154-5.

## Introduction

Immunohistochemistry is a powerful tool for studying neuronal tissue from humans at the molecular level and this is the foundation for a better understanding of neuronal function. Typically, human neuronal tissue is obtained *postmortem* from organ donors or body donors who donated their bodies to anatomical departments. The use of fresh neuronal tissue from organ donors for research is limited and due to ethical reasons often prohibited. In contrast, tissue from body donors can be obtained much easier because body donors have already given written consent during a lifetime that his/her body is dedicated to teaching and scientific purposes. For this reason, neuronal tissue from body donors should be tested as an alternative source for immunohistochemical studies.

A prerequisite for immunohistochemical analyses is optimal tissue quality. To avoid loss of quality, it is recommended to refrigerate bodies after death and keep the time between death and the start of tissue fixation (*postmortem* interval) as short as possible (Werner et al. 2000; Waldvogel et al. 2006; Almulhim and Menezes 2022). However, such a controlled procedure is very challenging, and only possible for donors who die in an intensive care unit.

People who donate their bodies to anatomical departments often die outside a monitoring facility and as there is uncertainty about the exact time of death, inaccuracies, and variability in *postmortem* interval estimates are unavoidable. Additionally, there is uncertainty about the ambient temperature bodies are exposed to until they are transferred to anatomical departments. Although neuronal tissue (peripheral nerves) from body donors has been analyzed by immunohistochemistry (Rein et al. 2013, 2015, 2020; Blumer et al. 2016; Pascual-Font et al. 2016; Garcia-Mesa et al. 2017; Lienbacher et al. 2019), it would be crucial to define a period after death during which accurate immunohistochemistry is possible in body donors. Such information is currently lacking but would be relevant for scientists who analyze nerve tissue from body donors at the molecular level.

In the present study, we harvested spinal cord tissue samples from body donors as they routinely arrive at body donor facilities at 24, 48, and 72 h *postmortem* intervals. We used a series of general and specific neuronal markers as well as glial markers and evaluated the stability of multi-color immunofluorescence and horseradish peroxidase (HRP) immunohistochemistry*.* Results in body donor spinal cords were compared with immunolabeling assays in fresh spinal cord obtained 2 h *postmortem* from an organ donor. We demonstrate that 24 h after death, different neuronal elements can be identified with high fidelity in the human spinal cord by immunofluorescence/HRP immunohistochemistry and high contrast images can be used for accurate computer-based quantitative analyses. With prolonged *postmortem* intervals (48 and 72 h), the staining capacity of the antibodies gradually decreases in an antibody-specific fashion.

## Materials and methods

### Tissue collection and preparation

From 15 body donors, the cervical segments 4–6 of the spinal cord were collected and analyzed. Spinal cords were from voluntary body donors of both sexes (seven females and eight males) who provided written consent to donate their dead bodies to be used for teaching and research at the Center of Anatomy and Cell Biology, Medical University of Vienna. The age of the donors ranged between 58 and 103 years (mean and standard deviation 81.2 ± 13.08) and none of them had a history of neurological disorders. Once delivered to the Department of Anatomy, body donors were kept in a mortuary at 4 °C until dissection. For all body donors, the time of death was precisely known, and they were divided into three groups. Each group contained five subjects. In the first group, the time between death and start of tissue fixation (*postmortem* interval) was 24 h, in the second 48 h, and in the third group 72 h.

Following dissection, the cervical spinal cord was subdivided into three pieces, each piece corresponding to a single spinal cord segment. Then, the issue was immersion fixed by 4% paraformaldehyde (PFA) in 0.1 M phosphate buffer (PB, pH 7.4) for 24 h at 4 °C and afterward stored in phosphate-buffered saline (PBS) for further processing. In every group, handling procedures for tissue processing were the same.

To evaluate results in the spinal cord from body donors, we analyzed the spinal cord (thoracic segments) of an organ donor (39 years old) used in a previous study (Gesslbauer et al., 2017). The *postmortem* interval of the organ donor spinal cord was 2 h and because of this short *postmortem* interval, it was used as a control for all other studied groups. The study has been approved by the local ethics committee (protocol number of the approval No. 1213/2012 and 1373/2021).

### Tissue preparation and processing

Spinal cords from the organ donor and body donors were cryoprotected in graded concentrations of sucrose (10, 25, and 40%) in PBS containing 0.05% sodium azide to avoid bacterial and fungal contamination, frozen, and stored at − 80 °C according to our guidelines for cryo-embedding (Blumer et al. 2019). For histological and immunohistochemical evaluation, tissue was cryo-sectioned at 10-μm thickness.

### Histology

Human spinal cord sections were labeled with hematoxylin–eosin using a standard protocol (hematoxylin 3 min and eosin 1 min). Thereafter, sections were dehydrated in graded solutions of alcohol and coverslipped in DPX mounting medium (Merck/Millipore, Temecula, CA USA).

### Immunohistochemistry

#### Antibodies and antibody characterization

Different neuronal marker antibodies were used to visualize nervous elements in the spinal cord. This included pan-neuronal markers (anti-neurofilament, anti-synaptophysin, and anti-nuclear neuronal protein), specific neuronal markers (anti-calcitonin gene-related peptide, anti-choline acetyltransferase, and anti-vesicular acetylcholine transporter) and glial cell markers (anti-myelin basic protein). The primary antibodies used in the present study including working dilutions, RRID numbers, and suppliers are listed in Table 1.

**Table 1:** List of primary and secondary antibodies used in the present study including working dilution, RRID number, and suppliers

Primary antibodies	Working dilution	Catalog/RRID number	Supplier
General markers for axons/synapses/nerve cells			
Chicken anti-neurofilament	1:2000	AB5539/AB_177520	Merck/Millipore
Mouse anti-synaptophysin	1:300	MAB329/AB**_**95786	Merck/Millipore
Mouse anti-neuronal nuclear protein	1:500	1.1.1.1MAB377/AB_2298772	Merck/Millipore
Marker for putative nociceptor fibers			
Rabbit anti-CGRP	1:1000	14959/AB_672598	Cell signaling technology
Marker for myelin			
Rat anti-MBP	1:100	MAB386/AB_94975	Merck/Millipore
Markers for cholinergic axons/synapses			
Goat anti-ChAT	1:100	AB144P/AB_2079751	Merck/Millipore
Rabbit anti-VAChT	1:500	139 103/AB_887864	Synaptic systems

Fluorescent secondary antibodies	Working dilution	Catalog/RRID number	Supplier
Goat anti-chicken IgG AF568	1:500	A-11041/AB_2534098	Thermo Fisher Scientific
Goat anti-chicken IgG AF647	1:500	A-21449/AB_2535886	Thermo Fisher Scientific
Goat anti-mouse IgM AF488	1:500	A-21042/AB_141357	Thermo Fisher Scientific
Goat anti-mouse IgM rhodamine	1:200	AP128R/AB11214322	Merck/Millipore
Goat anti rabbit IgG AF488	1:500	A-11034/AB_2576217	Thermo Fisher Scientific
Goat anti-rat IgG AF488	1:500	A-11006/AB_141373	Thermo Fisher Scientific
Rabbit anti-chicken IgG rhodamine	1:200	SA1-9512/AB_1075135	Thermo Fisher Scientific
Rabbit anti-goat IgG AF488	1:500	A11078/AB_141838	Thermo Fisher Scientific
Biotinylated secondary antibodies	Working dilution	Catalog/RRID number	Supplier
Goat anti-rabbit IgG biotinylated	Prediluted	21537/AB_916336	Merck/Millipore
Rabbit anti-goat IgG biotinylated	1:500	65-6140/AB_2533969	Thermo Fisher Scientific

The neurofilament antibody (AB5539, Merck-Millipore) is a polyclonal antibody and is raised against the bovine neurofilament heavy chain. By immunofluorescence, it has been demonstrated that this antibody visualizes myelinated and non-myelinated axons in humans (Gesslbauer et al. 2017; Blumer et al. 2020) and non-human tissue (Ghilardi et al. 2011; Tereshenko et al. 2021).

The monoclonal mouse antibody against the nuclear neuronal protein (NeuN, MAB377, Merck-Millipore) labels cell nuclei in neurons and is the most widely used neuronal marker in neuroscience research and neuropathological assays (Gusel'nikova and Korzhevskiy 2015).

The polyclonal goat antibody against choline acetyltransferase (ChAT, AB144P, Merck-Millipore) is raised against human placental ChAT. The antibody was validated in the human brain by pre-adsorption (Mesulam et al. 1992). By immunohistochemistry, it has been shown that this antibody visualizes cholinergic neurons in the human spinal cord (Gesslbauer et al. 2017) and brain (Lienbacher et al. 2019).

The monoclonal anti-synaptophysin antibody clone SP15 (MAB329, Merck-Millipore) is validated for use in ELISA, Western blot, and immunofluorescence for the detection of the synaptic vesicle protein synaptophysin (manufacturer’s datasheet).

The monoclonal antibody against MBP (MAB386) is raised against bovine myelin basic protein and was tested by Western blot in mouse brain (manufacturer’s datasheet). This antibody has been shown to visualize myelinated axons in the human spinal cord (Blumer et al. 2020) and non-human tissue (Nakanishi et al. 2005).

The monoclonal antibody against calcitonin gene-related peptide (CGRP, 14959, Cell Signaling) visualizes CGRP in the central and peripheral nervous system. This antibody was validated in carcinoma patients exhibiting a correlation between perineural invasion and lymph node metastasis (Zhang et al. 2021).

The VAChT antibody (139 103 Synaptic system) is a polyclonal antibody and raised the against vesicular acetyl choline transporter, which is an integral protein of synaptic vesicles. This antibody was knockout-validated in mouse brains (Kljakic et al. 2021) and used to visualize cholinergic neurons in the human pancreas (Campbell-Thompson and Tang 2021).

#### Immunofluorescence

We performed one single, four double, and three triple-immunofluorescence stainings. Spinal cord sections were single-labeled with anti-neurofilament, double-labeled with anti-neurofilament plus (1) anti-ChAT, (2) anti-synaptophysin, (3) anti-CGRP, and (4) anti-NeuN, and triple-labeled using anti-neurofilament plus (5) anti-synaptophysin/anti-CGRP, (6) anti-synaptophysin/anti-VAChT, and (7) anti-CGRP/anti-MBP. In staining combination (4), we used bisbenzimide (Hoechst 33342, Thermo Fisher Scientific) to counterstain cell nuclei.

Before immunolabeling, sections were air-dried for 30 min. Then they were blocked for 1 h with 10% normal rabbit serum (staining combination 1) or 10% normal goat serum (staining combinations 2–7) followed by incubation with the primary antibodies diluted in PBS containing 0.1% Triton X (PBS-T) for 48 h at 4 °C. After washing in PBS, sections were incubated with the secondary antibodies that were conjugated with different fluorochromes including Alexa Fluor (AF) 488, AF 568, and AF 647. The incubation time for the secondary antibodies was 2 h at 37 °C. Thereafter, sections were rinsed again in PBS and coverslipped with fluorescence mounting medium from Dako (DakoCytomation, Santa Clara, CA, USA). The fluorochrome conjugated secondary antibodies including working dilutions, RRID numbers, and supplier are listed in Table 1.

#### Horseradish peroxidase immunohistochemistry

HRP immunohistochemistry was performed in ChAT and CGRP staining. Sections were pretreated with 1% H_2_O_2_ to suppress the endogenous peroxidase activity following incubation with 10% normal rabbit serum (ChAT staining) or 10% goat serum in PBS-T (CGRP staining). Thereafter, sections were incubated with the primary antibodies as described above. After washing in PBS, sections were incubated for 2 h at 37 °C with the biotinylated secondary antibodies rabbit anti-goat for the ChAT staining or goat anti-rabbit for the CGRP staining. After a further washing step, sections were incubated with avidin-neutravidin conjugated with HRP for 2 h at 37 °C. The antigenic site was visualized using 0.01% diaminobenzidine (DAB) containing 0.06% H_2_0_2_ in 0.1 PBS for 5 to 10 min. Sections were coverslipped with aqueous mounting medium (Dako Cytomation). The biotinylated secondary antibodies including working dilutions, RRID numbers, and suppliers are listed in Table 1.

As a positive control, sections from the organ donor spinal cord were included in every staining combination and staining run. This approach allowed us to verify the validity of the staining process and exclude misinterpretation of results in the body donor spinal cord**.** Omission of the primary antibodies and using the secondary antibodies alone resulted in a complete lack of immunostaining. This confirmed that the secondary antibodies bind specifically to the primary antibodies.

### Microscopic imaging protocol

#### Light microscopy

Hematoxylin–eosin staining sections were analyzed with a slide scanner microscope (Olympus VS120, Olympus Europa SE & Co. KG, Hamburg, Germany).

Sections labeled by the HRP immunohistochemistry technique were examined with a light microscope (Axioscope, Zeiss, Jena, Germany) and photo-documented using a digital camera (Nikon Eclipse 600 camera, Nikon, Tokyo, Japan) connected to the microscope.

#### Confocal laser scanning microscopy

Fluorescently labeled sections were analyzed with a confocal laser scanning microscope (CLSM Olympus FV3000). Images were captured with dry lenses of 4x and 20x magnification and oil lenses of 60x magnification. A series of virtual CLSM sections of 1-µm thickness were cut through the structures of interest. Each section was photo-documented with a 1024 × 1024 pixel resolution and 3D projections were rendered using ImageJ software (NIH, Bethesda, MD, USA). Single-colored images (Fig. 2) were generated using a laser with excitation wavelength 633. Double-colored images (Figs. 3, 4, 5, 6, 7, 8, 9) were generated using lasers with excitation wavelengths of 488 and 568 nm, and triple-colored images (Figs. 10, 11, 12) using additionally a laser with excitation wavelengths of 405 or 640 nm. In certain cases, bright-field images were recorded in the CLSM to correlate immunolabeled structures to morphological structures (Fig. 5a’’, b’’, c’’, d’’).

#### Scanning strategy

In all staining combinations and all *postmortem* groups, fluorescently labeled sections were analyzed and photo-documented immediately (within 1 day) after the staining experiments were completed. For every staining combination, individual CLSM settings were manually adjusted and care was taken to avoid oversaturation or undersaturation of images. Once found, settings parameters were kept constant for image acquisition of all samples in each staining combination and *postmortem* interval. This scanning strategy allowed us to determine changes in the signal intensity at different *postmortem* intervals.

### Histological quantification

#### Axon counts

To evaluate the accuracy of our immunofluorescence, we separately counted neurofilament positive axons and neurofilament/ChAT-positive axons in the anterior roots of the human spinal cord (*N* = 3). This was done in the spinal cords at 24 and 48 h *postmortem* intervals. Whole cross sections of the anterior roots were scanned with a slide scanner [Axio Observer Z.1 microscope (Carl Zeiss Meditec AG, Jena, Germany)] followed by automated counts of axons using StrataQuest version 5.1.249 and Tissue Quest version 4.0.1.0128 (TissueGnostic Vienna, Austria).

#### Quantification of the staining intensity

The intensity of the antibody signals was quantified in human spinal cords with 2-h (*N* = 1), 24-h (*N* = 3), 48-h (*N* = 3), and 72-h (*N* = 3) *postmortem* intervals. The antibodies studied in each *postmortem* interval were anti-neurofilament, anti-ChAT, anti-CGRP, and anti-synaptophysin. For the quantification of the staining intensity, images of the specimens were captured at 20x magnification using identical CLSM settings. In each image, 25 randomly selected squares of 6 × 6 μm were used to measure the intensity of the antibodies signal (i.e., optical density). In all cases, for background correction, ten optical density values of the same area were taken. Then, the background value was subtracted, and the values of the antibody optical density were normalized with respect to the 2-h *postmortem* spinal cord. The antibody staining intensity was set at 100% in the 2-h *postmortem* spinal cord and values for spinal cords with 24-, 48-, and 72-h *postmortem* intervals were calculated as the percentage of the 2-h *postmortem* control value.

#### Statistical analyses

The axon quantification data of double-positive axons (ChAT and neurofilament) and single-positive neurofilament axons were statistically compared using Student’s *t* test at a level of significance of *P* < 0.05. The data of staining intensity (anti-neurofilament, anti-ChAT, anti-CGRP, and anti-synaptophysin) of the different *postmortem* intervals (2, 24, 48, and 72 h) were statistically compared using the one-way-ANOVA test followed by post hoc multiple comparisons (Holm–Sidak method) at a significance level of *P* < 0.05. All values were expressed as the mean ± standard error of the mean (SEM). Statistical analysis was carried out in Sigma Plot, version 11 (Systat Software, San José, CA, USA).

## Results

### Histology

**Fig. 1:**
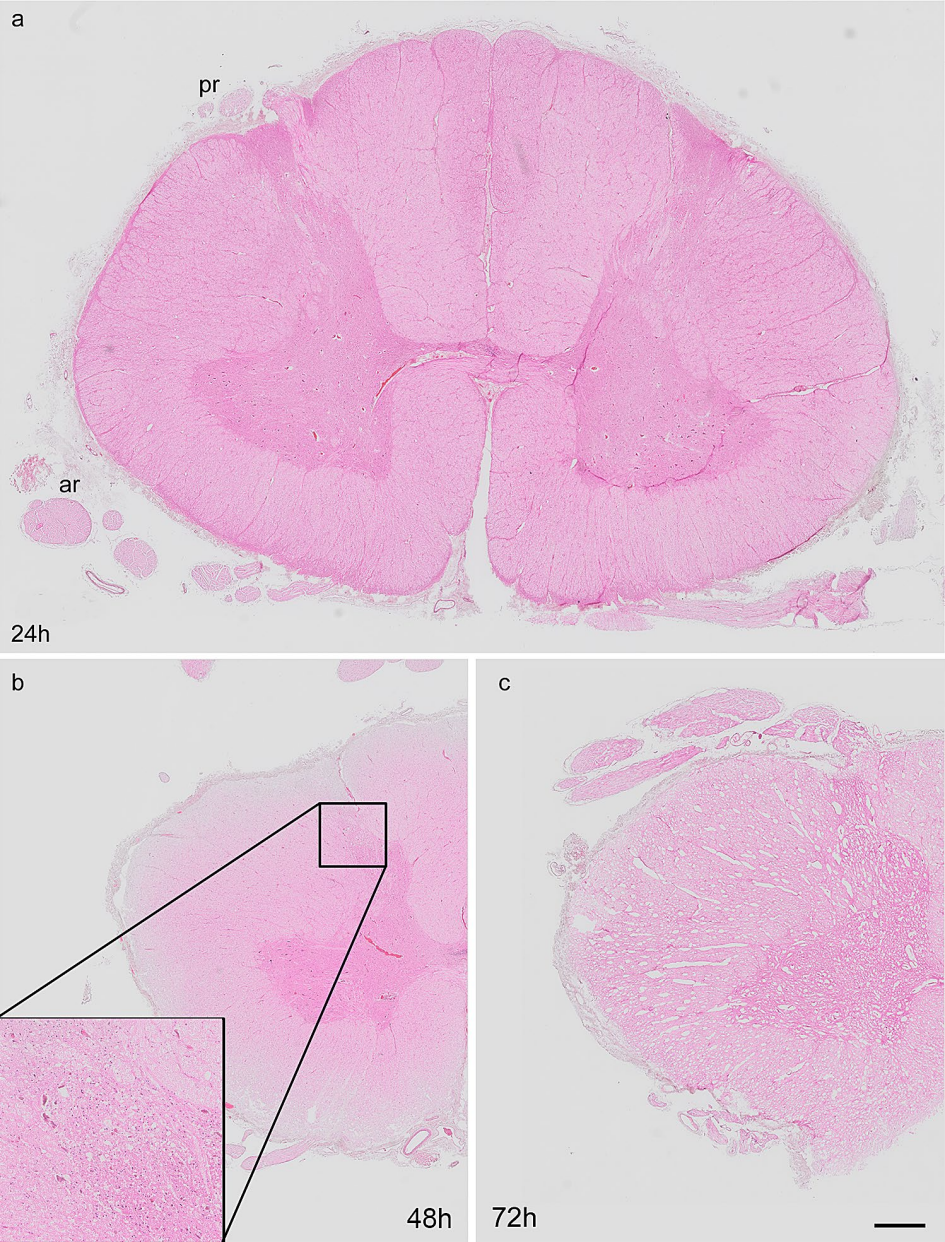
Hematoxylin–eosin staining of human spinal cords after different *postmortem* intervals. **a** Cross section of the spinal cord at 24 h *postmortem*. The structure of the spinal cord appears normal without observable histological changes. Anterior roots (ar), posterior roots (pr). **b**, **c** Half of the spinal cord after 48 h (**b**) and 72 h (**c**) *postmortem* interval. At time point 48 h, structural defects including small gaps (inset in **b**) are present in the spinal cord whereas at 72 h, the gray and white matter exhibit large gaps, indicating advanced autolysis of the tissue (**c**). Scale bar 1000 μm (**a**–**c**)

The histology of the spinal cord from body donors was analyzed using hematoxylin–eosin staining. The spinal cord at 24-h *postmortem* interval exhibited strong staining and the tissue architecture appeared normal. The two parts of the spinal cord, the inner H-shaped gray matter, and the outer white matter were clearly identifiable (Fig. 1a). In line with a previous study, we observed that the anterior horn of the gray matter has a wide lateral expansion at the cervical level of the spinal cord (Kameyama et al. 1996). Forty-eight hours after death, small gaps were observed readily in the white and gray matter of the spinal cord (Fig. 1b) and they increased in size and number 72 h *postmortem*, indicating advanced autolysis of the tissue (Fig. 1c). Moreover, 72 h *postmortem* the staining intensity decreased and there was no sharp boundary between the spinal cord’s gray and white matter.

### Lipofuscin autofluorescence

**Fig. 2:**
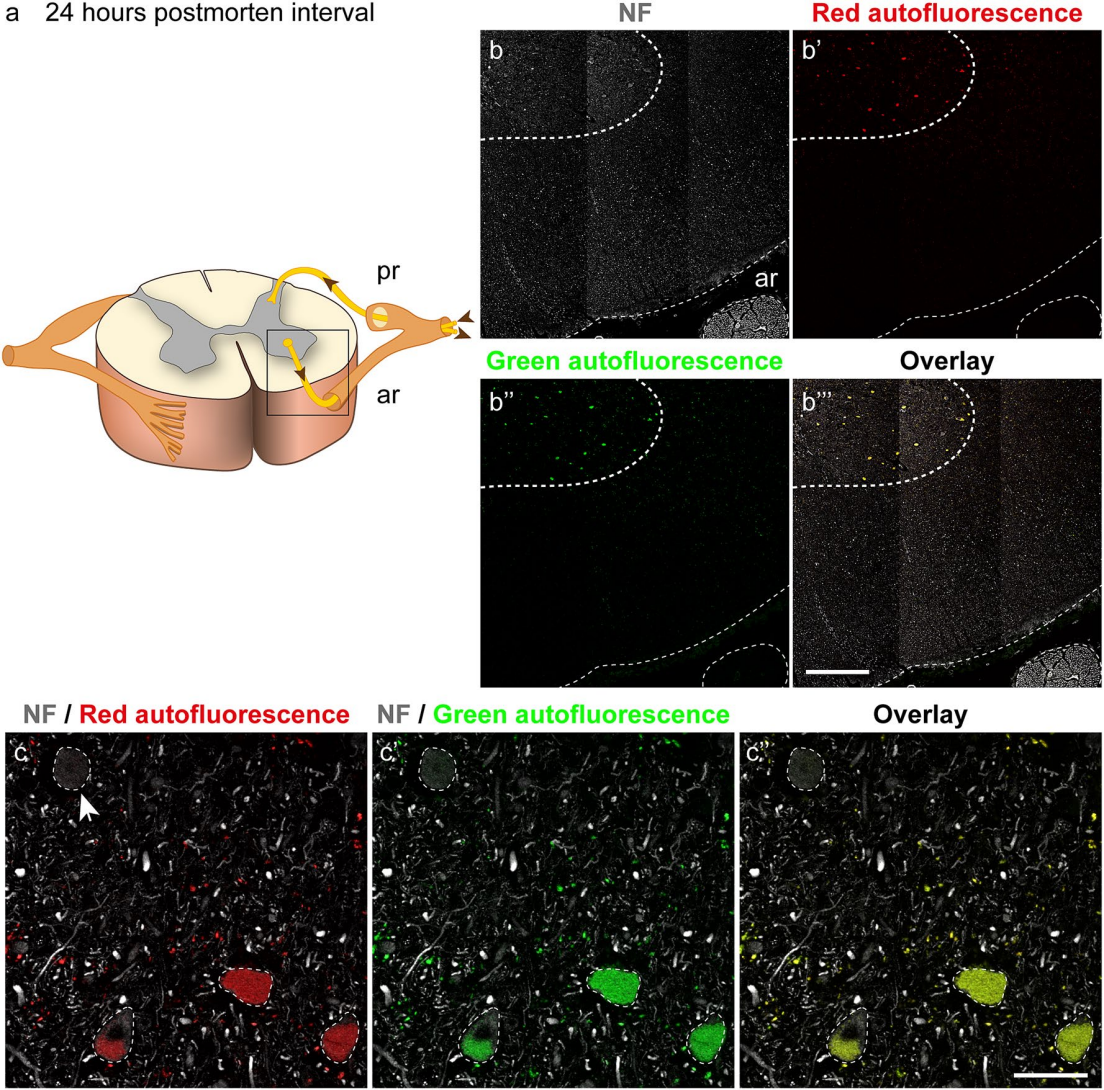
Human spinal cord labeled with anti-neurofilament (NF) after 24 h *postmortem* interval showing lipofuscin autofluorescence. **a** Scheme with an inset illustrating the part of the spinal cord shown in figure **b–b’’’**. Posterior root (pr), anterior root (ar). **b–b’’’** Spinal cord in neurofilament staining (**b**), red autofluorescence (**b’**), green autofluorescence (**b’’**), and overlay (**b’’’**). Thethick dashed line demarcates the gray and white matter and thethin dashed lines the surface of the spinal cord and the anterior root. **b** Grey and white matter and the anterior root (ar) express neurofilament. **b’** Motoneurons of the anterior horn exhibit red and **b’’** green autofluorescence due to lipofuscin deposits. **b’’’** The overlay of **b–b’’** shows that the mixture of red and green autofluorescence results in yellow color. **c–c’’** High magnification of motoneurons of anterior horn demarcated with a dashed line showing neurofilament signal and red lipofuscin autofluorescence (**c**), neurofilament and green lipofuscin autofluorescence (**c’**), and the overlay of **c–c’** (**c’’**). The amount of lipofuscin varies and two cell bodies are fully covered with lipofuscin, one cell partly, and one cell (arrow) is free of lipofuscin. Lipofuscin granules exhibiting red and green autofluorescence are also visible outside the cell bodies in the neuropil.Scale bars 500 µm in **b’’’** for **b–b’’’** and 50 µm in **c’’** for **c–c’’**

Lipofuscin is a lipid-containing degradation product and increases with age in neuronal and non-neuronal tissue (Brody 1960; Shiers et al. 2021). In the human brain, lipofuscin deposits were observed intra- as well as extracellularly (Waldvogel et al. 2006). Although lipofuscin is virtually present in every type of neuron, it is most abundant in neurons initiating movements including motoneurons of the spinal cord (Gray and Woulfe 2005). Typically, lipofuscin exhibits strong red and green autofluorescence and can be recognized in a fluorescence microscope when using appropriate filters (Gray and Woulfe 2005). Concordant with this, we observed high lipofuscin autofluorescence in the motoneurons of the anterior horn of the organ donor and body donor spinal cords. Due to the older age, the lipofuscin accumulation was higher in body donors (mean age 81.2 ± 13.08 years) than in the 39-year-old organ donor. The inset in Fig. 2a highlights the anterior segment of the spinal cord, which is shown in immunofluorescent staining in Fig. 2b’–b’’’. Figure 2c–c’’ visualizes lipofuscin inside motoneuron cells bodies and processes in the neuropil. In motoneurons, the lipofuscin accumulation was graded and while few motoneuron cell bodies lacked lipofuscin, most cell bodies were filled to various degrees with this age-related cellular inclusion (Fig. 2c–c’’). Because of the lipofuscin autofluorescence, care is necessary when interpreting immunofluorescence staining in the spinal cord and, at appropriate places, we referred to this point in the manuscript.

### Double immunofluorescence and peroxidase immunohistochemistry

**Fig. 3:**
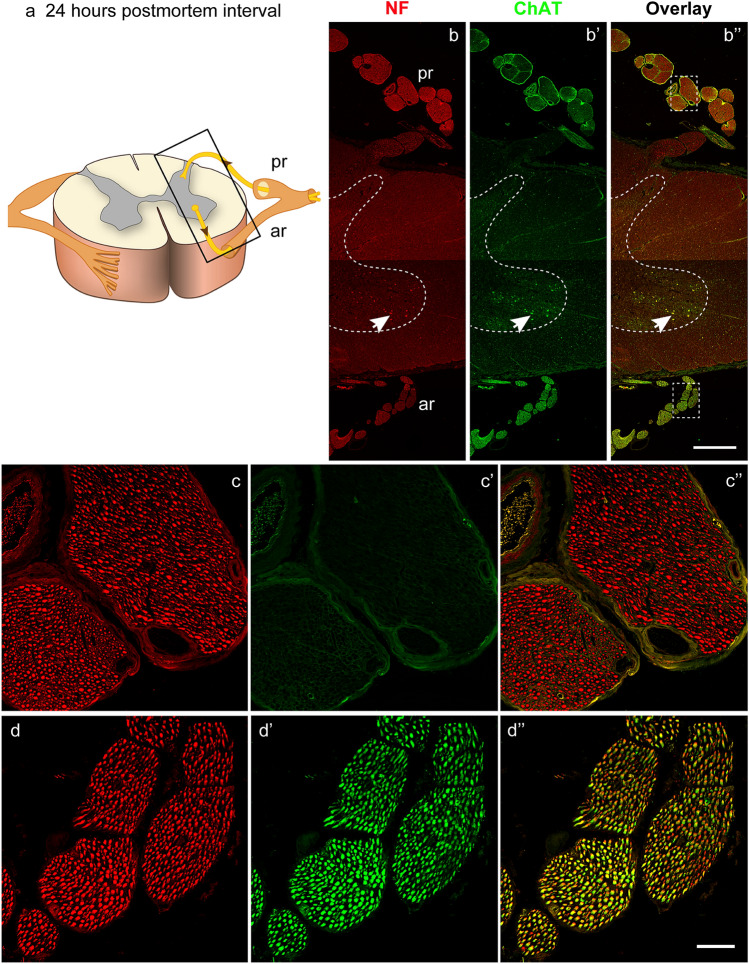
Double labeling with anti-neurofilament (NF) and anti-ChAT after 24 h *postmortem* interval. **a** Scheme with an inset illustrating the part of the spinal cord shown in figure **b–b’’**. Posterior roots (pr), anterior roots (ar). **b** The spinal cord in neurofilament staining. The gray and white matter and the anterior (ar) and posterior (pr) roots express neurofilament. Thedashed line demarcates the gray and white matter. Motoneurons of the anterior horn exhibit red autofluorescence due to lipofuscin deposits (arrow). **b’** The spinal cord in ChAT staining. Fascicles of the anterior root exhibit ChAT immunoreactivity whereas fascicles of the posterior root lack ChAT immunoreactivity. Thegreen pial contour covering the surface of the posterior root represents an artificial signal. Motoneurons of the anterior horn exhibit green autofluorescence due to lipofuscin (arrow). **b’’** Overlay of **b** and **b’**, shows that the nerve fascicles of the anterior root are positive for neurofilament and ChAT. **c–c’’** The fascicles of the posterior root (inset in the upper part of **c’’**). Axons of the posterior root are neurofilament positive (**c**) but lack ChAT (**c’**). In the overlay (**c’’**), neurofilament and ChAT staining is merged. **d–d’’** Fascicles of the anterior root (inset in the lower part of **b’’**). Axons of the anterior root express neurofilament (**d**) and ChAT (**d’**). In the overlay **d’’**, the mix of the red neurofilament and green ChAT signal results in yellow color.Scale bars: 1000 µm in **b’’** for **b–b’’**; 100 µm in **d’’** for **c–c’’** and **d–d’’**

We performed four double-labeling immunofluorescence assays in the spinal cord of the organ donor and body donors at different *postmortem* intervals. Double immunofluorescence included labeling with anti-neurofilament plus (1) anti-ChAT, (2) anti-synaptophysin, (3) anti-CGRP, and (4) anti-NeuN. Alternatively, HRP immunohistochemistry for anti-ChAT and anti-CGRP was performed. Anti-ChAT is a marker for cholinergic neurons, anti-synaptophysin is a marker for synapses, anti-CGRP is a marker for putative nociceptor fibers, and anti-NeuN is a marker for the nuclei of neuronal cells. In our immunolabeling assays, some antibodies exhibited stronger signal intensity than others and we therefore adapted the CLSM settings for every individual staining combination. As a result of this approach, the red and green lipofuscin autofluorescence is visible in some figures (Figs. 2b’–b’’’, c–c’’, 3b–b’’, 6a–a’’, b–b’’, 9b–b’’) whereas in other figures only red autofluorescence (Figs. 7c, c’’, e, e’’, 8a, 10b–b’’, c–c”) or almost no autofluorescence is visible (Figs. 10d–d”, 11e–e”, 11a, 12a–a’’, b–b’’). A supplementary figure (Supplementary Fig. 1) is provided showing autofluorescence signal dependent on CLSM settings.

**Fig. 4:**
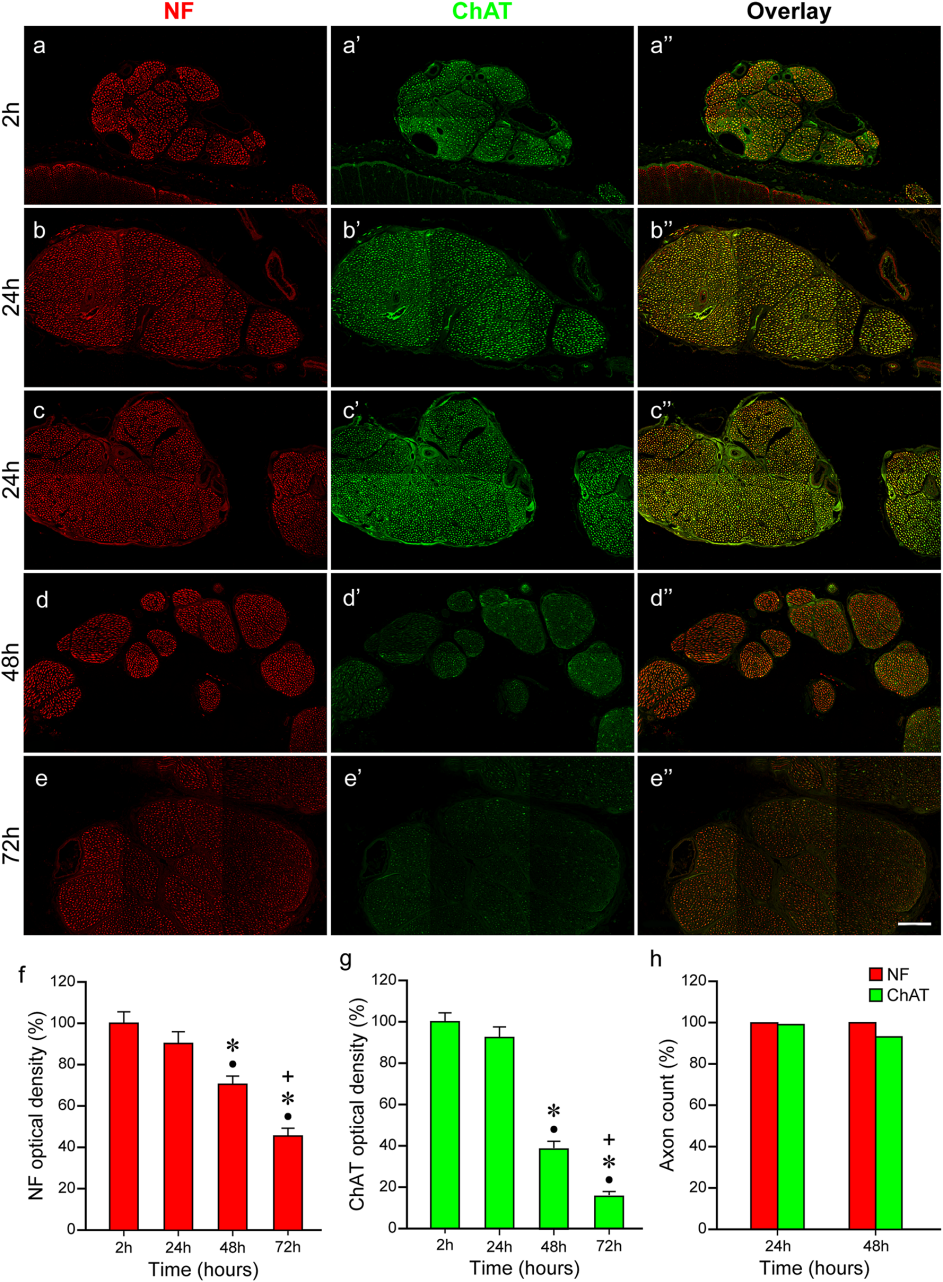
Immunolabeling and quantitative analyses of the anterior roots of the organ donor and body donor spinal cord. **a–a’’**, **b–b’’**, **c–c’’**, **d–d’’**, **e–e’’** Labeling with anti-neurofilament (NF) and anti-ChAT of the 2-h organ donor spinal cord (**a–a’’**), the body donor spinal cord from two different subjects 24 h (**b–b’’**, **c–c’’**), and from one subject 48 h (**d–d’’**), and 72 h *postmortem* (**e–e’’**). A reduction in the signal intensity is observed in the neurofilament (**a**, **b**, **c**, **d**, **e**) and ChAT staining (**a’**, **b’**, **c’**, **d’**, **e’**) although the degree varies. In the neurofilament staining (**a**, **b**, **c**, **d**, **e**), the brightness of the signal is similar at time point 2 h (**a**), 24 h (**b** and **c**), and 48 h (**d**) and appears apparently reduced not until 72 h (**e**). A stronger signal reduction is observed in the ChAT staining (**a’**, **b’**, **c’**, **d’**, **e’**). At time point 2 h (**a’**) and 24 h (**b’** and **c’**), the ChAT intensity is almost equal, lower at time point 48 h (**d’**), and lowest at 72 h (**e’**). Overlays of the neurofilament and ChAT staining are shown in **a’’**, **b’’**, **c’’**, **d’’**, **e’’**. The overlay in **a’’**, **b’’**, and **c’’** appears in yellow color due to the mix of the strong red neurofilament (**a**, **b**, **c**) and green ChAT signal (**a’**, **b’**, **c’**). Overlays in **d’’** and **e’’** appear inred–orange color due to the stronger decrease of the green ChAT signal (**d’**, **e’**) relative to the red neurofilament signal (**d**, **e**).Scale bar: 100 µm in **e’’** for **a–a’’**, **b–b’’**, **c–c’’**, **d–d’’**, and **e–e’’**. **f**, **g** Bar chart illustrating the change of the neurofilament and ChAT staining intensity 2, 24, 48, and 72 h *postmortem*. At time point 2 h, the neurofilament and ChAT staining intensity is set at 100% and values for 24, 48, and 72 h are presented as a percentage of the 2 h reference value (**f**: neurofilamentred bars, **g**: ChATgreen bars). At 24 h, the brightness of the neurofilament is at about 90% (**f**: second bar), at 48 h about 70% (**f**: third bar), and at 72 h to about 45% (**f**: fourth bar). In the ChAT staining, the signal reduction is minimal 24 h postmortem and 93% of the 2 h value (**g**: second bar). Signal reduction is to about 40% at time point 24 h, (**g**: third bar), and to about 20% at 72 h (**g**: fourth bar).Black dots (●) indicate significant differences with time point 2 h,asterisks (*) indicate significant differences with time point 24 h, andplus signs (+) mark significant differences with time point 48 h (one-way ANOVA test followed by Holm–Sidak post hoc method, *P* < 0.05). (**h**) Percentage of neurofilament and ChAT-positive axons in the anterior root after 24 h and 48 h *postmortem* interval. At 24 h, the number of neurofilament and ChAT-positive axons is almost the same proving that all neurofilament positive axons in the anterior root express ChAT as well. At 48 h, a higher number of neurofilament than ChAT-positive axons is counted because, in some axons, ChAT signals are too low for being detected by computer-based quantification. (*t* test; *P* < 0.05; *N* = 3 for neurofilament and ChAT)

**Fig. 5:**
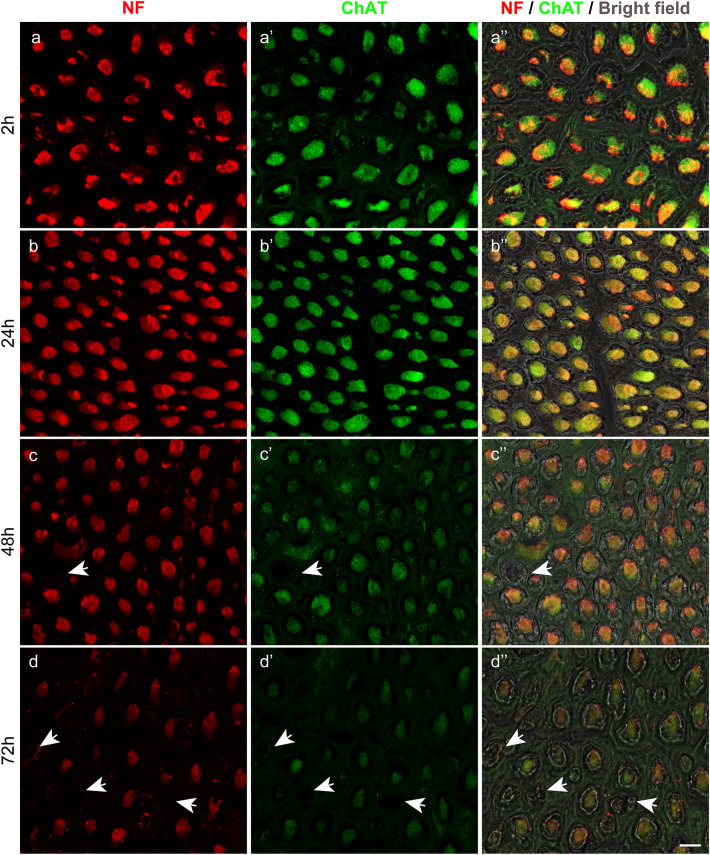
High-resolution images of immunolabeled anterior root axons. **a–a’**’ Axons from the 2-h organ donor spinal cord and **b–b’’**, **c–c’’**, **d–d’’** from the body donor spinal cord at different *postmortem* intervals. Axons are shown in anti-neurofilament (NF, **a**, **b**, **c**, **d**), anti-ChAT staining (**a’**, **b’**, **c’**, **d’**) and overlays (**a’’**, **b’’**, **c’’**, **d’’**). At time point 2 and 24 h, all axons exhibit strong neurofilament (**a**, **b**) and ChAT (**a’**, **b’**) signals and there is a complete overlap of neurofilament and ChAT signals (**a’’**, **b’’**). Most axons are of large diameter (putative α-motoneurons) and few are of small diameter (putative γ-motoneurons). In the overlay **a’’**, **b’’**, fluorescence and bright-field images are merged showing that all axons are covered with a myelin sheath. At time point 48 h (**c–c’’**), neurofilament (**c**) and ChAT signals (**c’**) decrease but the reduction is stronger in ChAT staining. Some axons (arrow) lack antibody signals. At time 72 h (**d–d’’**), a further signal reduction is visible, and signals lose sharpness and homogeneity. The number of axons without antibody signal (arrows) has increased. Additionally, axons are more separated from each other.Scale bar: 10 µm in **d’’** for **a–a’’**, **b–b’’**, **c–c’’**, and **d–d’’**

Following anti-neurofilament/anti-ChAT incubation, neurofilament signals were observed in the gray and white matter of the spinal cord as well as in the anterior and posterior roots. ChAT signals were present in the anterior but not in the posterior roots of the spinal cord. The inset in Fig. 3a highlights the segment of a 24-h body donor spinal cord, which is immunolabeled in Fig. 3b–b’’. Images of the posterior root (Fig. 3c–c’’) and anterior root (Fig. 3d–d’’) are provided at higher resolution; 24-h *postmortem,* neurofilament, and ChAT signals were strong and almost equal to those observed in the spinal cord of the organ donor (Figs. 4a–a’’, b–b’’, c–c’’, 5a–a’’, b–b’’). We combined fluorescence and bright-field images to match fluorescently labeled structures with anatomical structures. By this technique it was possible to visualize the myelin sheath surrounding the fluorescently labeled axons of the anterior root (Fig. 5a’’, b’’).

After 48- and 72-h *postmortem* intervals, the neurofilament and ChAT signals decreased. The signal reduction was moderate in the neurofilament but strong in the ChAT staining as demonstrated for the anterior roots (Fig. 4d–d’’, e–e’’, low resolution; Fig. 5c–c’’ and d–d’’, high resolution). Forty-eight hours after death, few axons completely lacked neurofilament/ChAT signals, but this was more frequent 72-h *postmortem* (arrows in Fig. 5c–c’’, d–d’’). Additionally, by 72 h, most axons revealed less clear delineation coupled with incomplete labeling (Fig. 5d–d’’).

We quantified the staining intensity in the 2-h *postmortem* spinal cord of the organ donor (*N* = 1) and in the spinal cords of the body donors at every *postmortem* interval (*N* = 3 for each *postmortem* interval). Measurements are percentages relative to the 2-h spinal cord value, which was the reference value and set at 100% (Fig. 4f, left bar; 4 g left bar). At time point 24 h, the neurofilament staining intensity was 90% of the 2-h reference value (Fig. 4f, second bar). At the time point of 48 h, the neurofilament signal was at about 70% (Fig. 4f, third bar) and at 72 h at around 45% of the reference value (Fig. 4f, fourth bar). Relative to the reference value, the ChAT signal was 93% at time point 24 h (Fig. 4g, second bar), 40% at time point 48 h (Fig. 4g, third bar), and less than 20% at 72 h (Fig. 4g, fourth bar).

We evaluated the precision of the neurofilament/ChAT immunolabeling by counting separately neurofilament and neurofilament/ChAT-positive axons in the anterior root of the 24- and 48-h spinal cord (*N* = 3 for each point of time). Because the anterior roots of the human spinal cord carry exclusively cholinergic (motor) axons, all neurofilament-positive axons in the anterior root should express ChAT as well. Automated quantification revealed that at time point 24 h, 99.5% of the axons in the anterior root expressed ChAT (Fig. 4h, left bars) and this was confirmed by high-resolution images, as there was a complete match of neurofilament/ChAT signals (Fig. 5b–b’’). The small gap to 100% could be explained that the ChAT signal varies along the axons (Zimmermann et al. 2013) and it is possible that axons with weaker ChAT signals were missed by the analysis software. At time point 48 h, significantly fewer ChAT-positive axons (90.3%) were counted (Fig. 4h, right bars). This finding proves that immunolabeling was very precise 24 h *postmortem* but no longer 48 h *postmortem*.

**Fig. 6:**
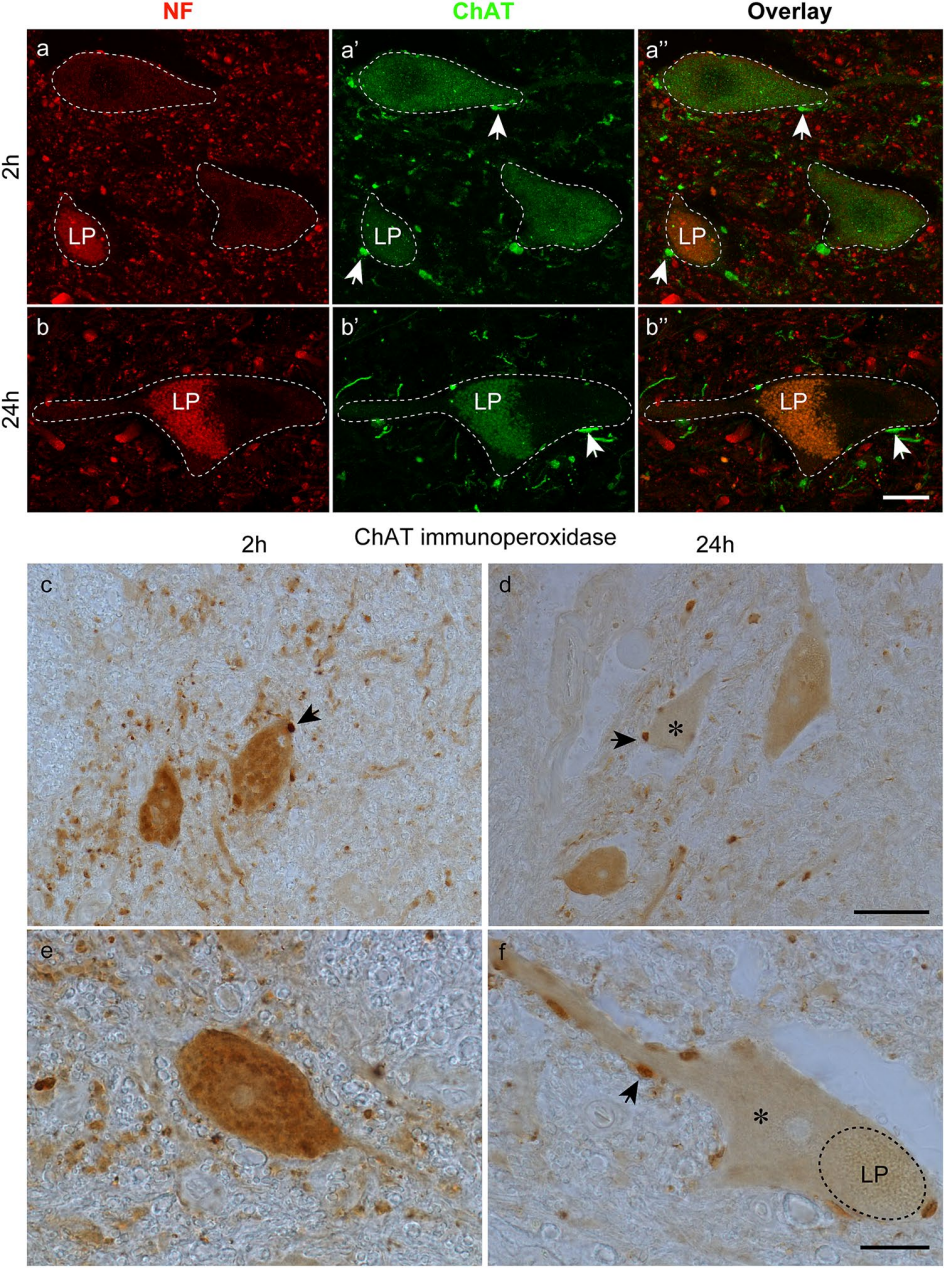
Immunofluorescence with anti-neurofilament (NF) and anti-ChAT, and HRP immunohistochemistry with anti-ChAT. **a–a’’**, **b–b’’** Immunofluorescence of motoneurons stained with anti-neurofilament (**a**, **b**) and anti-ChAT (**a’**, **b’**) from the organ donor (**a–a’’**) and 24 h body donor spinal cord (**b–b’’**).Dashed lines demarcate the motoneuron cell bodies (**a–a’’**) including the proximal dendrite (**b–b’’**). Some motoneurons exhibit red and green autofluorescence due to lipofuscin deposits (LP). A weak ChAT signal is observed 2 h *postmortem* (**a’**) but absent 24 h *postmortem* (**b’**). Synapses (arrows) contacting motoneuron cell bodies exhibit strong ChAT immunoreactivity (**a’**, **b’**). Overlays of the neurofilament (**a**, **b**) and ChAT (**a’**, **b’**) staining are presented in **a’’** and **b’’**.Arrows in **a’’**, **b’’** indicate ChAT-positive synapses. (**c–f**) Immunoperoxidase staining with anti-ChAT at lower (**c**, **d**) and higher magnification (**e**, **f**). At time point 2 h (**c**, **e**), the cell bodies exhibit a strong ChAT signal, which is weaker or absent (*, **d**, **f**) in cell bodies at time point 24 h (**d**, **f**). Lipofuscin is demarcated by adashed line at time point 24 h (LP, **f**). Synapses (some of them are indicated by* arrows*) with strong ChAT immunoreactivity cover the cell bodies and proximal dendrites of motoneurons (**c**, **d**, **f**).Scale bars: 20 µm in **b’’** for **a–a’’**, **b–b’’**, 50 µm in **d** for **c** and **d**, 20 µm in **f** for **e** and **f**

Although the ChAT signals were strong and almost equal in the motor axons 2-h and 24-h *postmortem*, ChAT immunoreactivity in motoneuron cell bodies was observed 2 h but not 24 h *postmortem* (Fig. 6a–a’’, b–b’’). Interestingly, the ChAT intensity was lower in motoneuron cell bodies than in motoneuron axons. It is important to note that the ChAT signal in motoneuron cell bodies was only visible in the green fluorescence but not in the red fluorescence channel of the CLSM (Fig. 6a–a’’), thus excluding being lipofuscin autofluorescence, which would be detectable in both fluorescence channels. We therefore conclude that the ChAT immunoreactivity in motoneuron cell bodies 2 h *postmortem* was specific. Different from that pattern, motoneuron cell bodies in the 24-h *postmortem* spinal cord exhibited signals in the green fluorescence and red fluorescence channel, indicating lipofuscin autofluorescence instead of a specific ChAT signal (Fig. 6b–b’’). Interestingly, cholinergic synapses, which were observed on motoneurons, exhibited bright ChAT signals 2 and 24 h *postmortem* (Fig. 6a’–a’’, b–b’’). The lack of ChAT signals in motoneuron cell bodies 24 h *postmortem* might be explained by the fact that the ChAT enzyme became dissociated or alternatively was at a level too low for detection by immunofluorescence. We therefore performed HRP immunohistochemistry because there are examples in the literature that this technique is more sensitive than immunofluorescence (Molne et al. 2005). By using this approach, we observed robust ChAT immunoreactivity in the motoneurons of the 2-h donor spinal cord (Fig. 6c, e), whereas in the 24-h body donor spinal cord, weak signals were found in some but not all motoneurons (Fig. 6d, f). Analog to immunofluorescence, cholinergic synapses exhibited a high signal intensity in immunoperoxidase staining (arrows in Fig. 6c, d, f).

**Fig. 7:**
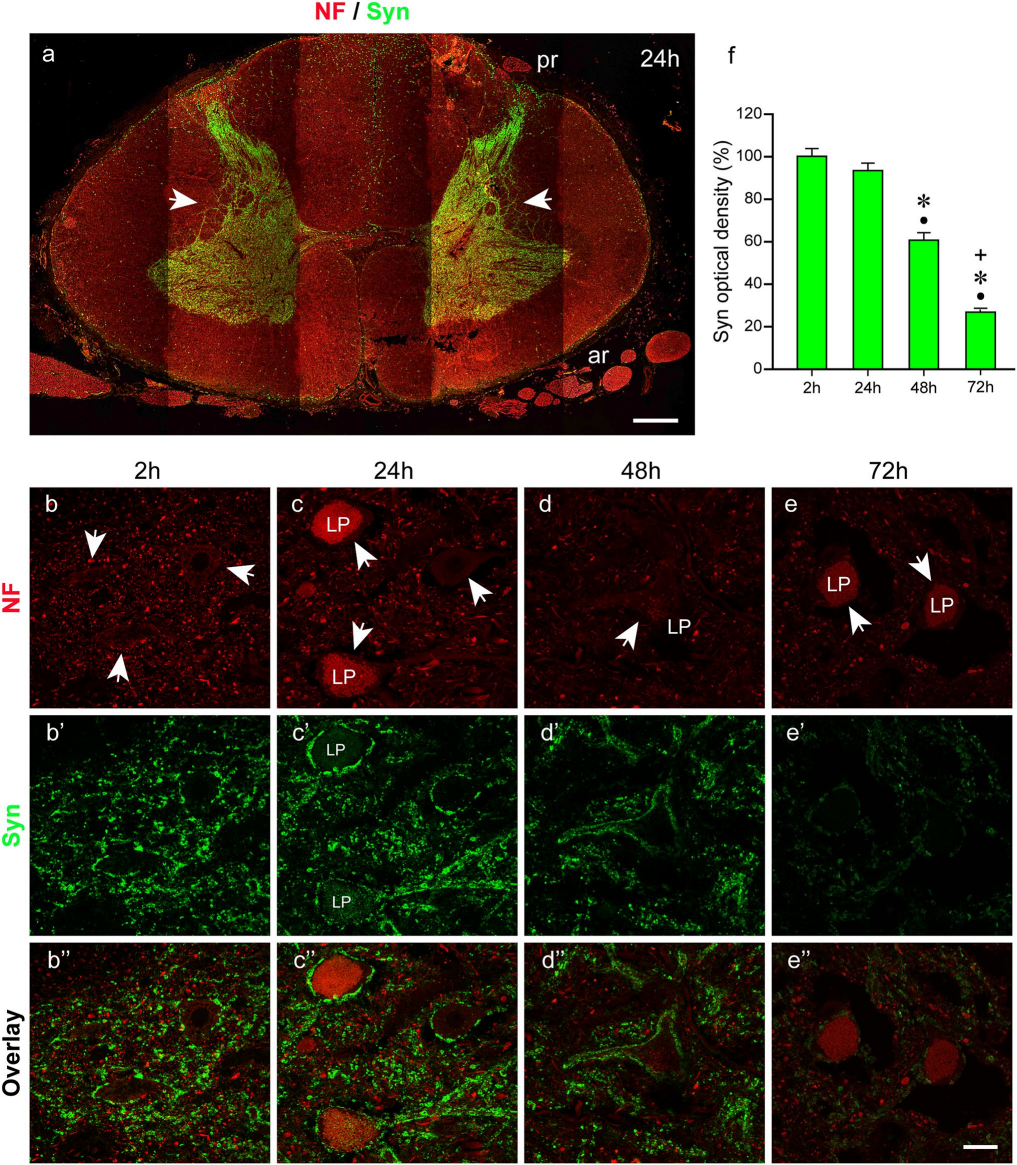
Double labeling with anti-neurofilament (NF) and anti-synaptophysin (Syn). **a** Cross section through a spinal cord 24 h *postmortem* illustrating synaptophysin immunoreactivity throughout the gray matter. Anterior roots (ar) and posterior roots (pr). Dendrites extending into the white matter are indicated byarrows. (**b–b’’**, **c–c’’**, **d–d’’**, **e–e’’**) Show anterior horn motoneuron cell bodies in neurofilament (**b**, **c**, **d**, **e**), synaptophysin staining (**b’**, **c’**, **d’**, **e’**), and overlay (**b’’**, **c’’**, **d’’**, **e’’**). Some motoneurons (arrows) exhibit red lipofuscin (LP) autofluorescence (**c**, **d**, **e**). At time points 2 h (**b–b’’)** and 24 h (**c–c’’**), the cell bodies including proximal dendrites of the motoneurons are fully covered with synaptophysin-positive synapses (**b’**, **b’’**, **c’**, **c’’**). At time point 48 h (**d–d’’**), synaptic density appears unchanged but the synaptophysin intensity is decreased (**d’**, **d’’**). At time point 72 h (**e–e’’**), the number of synapses is reduced and the synaptophysin signal (**e’**, **e’’**) is further reduced. Additionally, large gaps surround the cell bodies of the motoneuron. **f** Bar chart showing the reduction of the synaptophysin staining intensity. At the time point 24 h, the signal intensity is about 95% of the 2-h value (**f**, second bar**)**. At time point 48 h, the signal intensity is around 60% (**f**, third bar) and at 72 h about 25% (**f**, third bar) of the initial value (**f**, first bar).Black dots (●) indicate significant differences with time point 2 h,asterisks (*) indicate significant differences with time point 24 h, andplus signs (+) mark significant differences with time point 48 h. (one-way ANOVA test followed by Holm–Sidak post hoc method, *P* < 0.05).Scale bar: 1000 µm in **a**, 25 µm in **e’’** for **b–b’’**, **c–c”**, **d–d’’**, and **e–e’’**

We next performed labeling with anti-neurofilament and anti-synaptophysin. Synaptophysin is a protein present in synaptic contacts (De Camilli et al. 1988) and synaptophysin was observed throughout the spinal cord’s gray matter as demonstrated in a panoramic image of the body donor spinal cord 24 h *postmortem* (Fig. 7a). Synaptophysin was also found along cell processes (dendrites) extending from the gray into the white matter (*arrows* in Fig. 7a). We observed synaptophysin signals at the periphery of the spinal cord and in the posterior part of the spinal cord along the midline (Figs. 7a, 10a). Microscopic analyses showed that the fluorescence signal corresponded with plaques in the white matter.

To evaluate the quality of the synaptophysin labeling along *postmortem* intervals, we examined synaptic contacts on spinal cord motoneurons. We observed no differences between 2 and 24 h *postmortem*, and synapses expressing high levels of synaptophysin covered the surface of the motoneuron cell bodies (Fig. 7b–b’’, c–c’’). Forty-eight hours after death, the signal brightness was reduced but the synaptic density on motoneuron cell bodies appeared unchanged (Fig. 7d–d’’). At time point 72 h, the signal intensity further decreased and the synaptic density was reduced (Fig. 7e–e’’). Morphologically, large tissue-free gaps surrounded the motoneurons 72 h postmortem (Fig. 7e–e’’).

Measurements of the staining intensity demonstrated that the synaptophysin signal was almost equal in the 2-h *postmortem* spinal cord of the organ donor and in the body donor spinal cord 24 h *postmortem* (Fig. 7f)*.* At time point 48 h, the signal intensity of synaptophysin was reduced to about 60% of the 2-h value (Fig. 7f) and at time point 72 h to approximately 25% of the 2-h value (Fig. 7f).

**Fig. 8:**
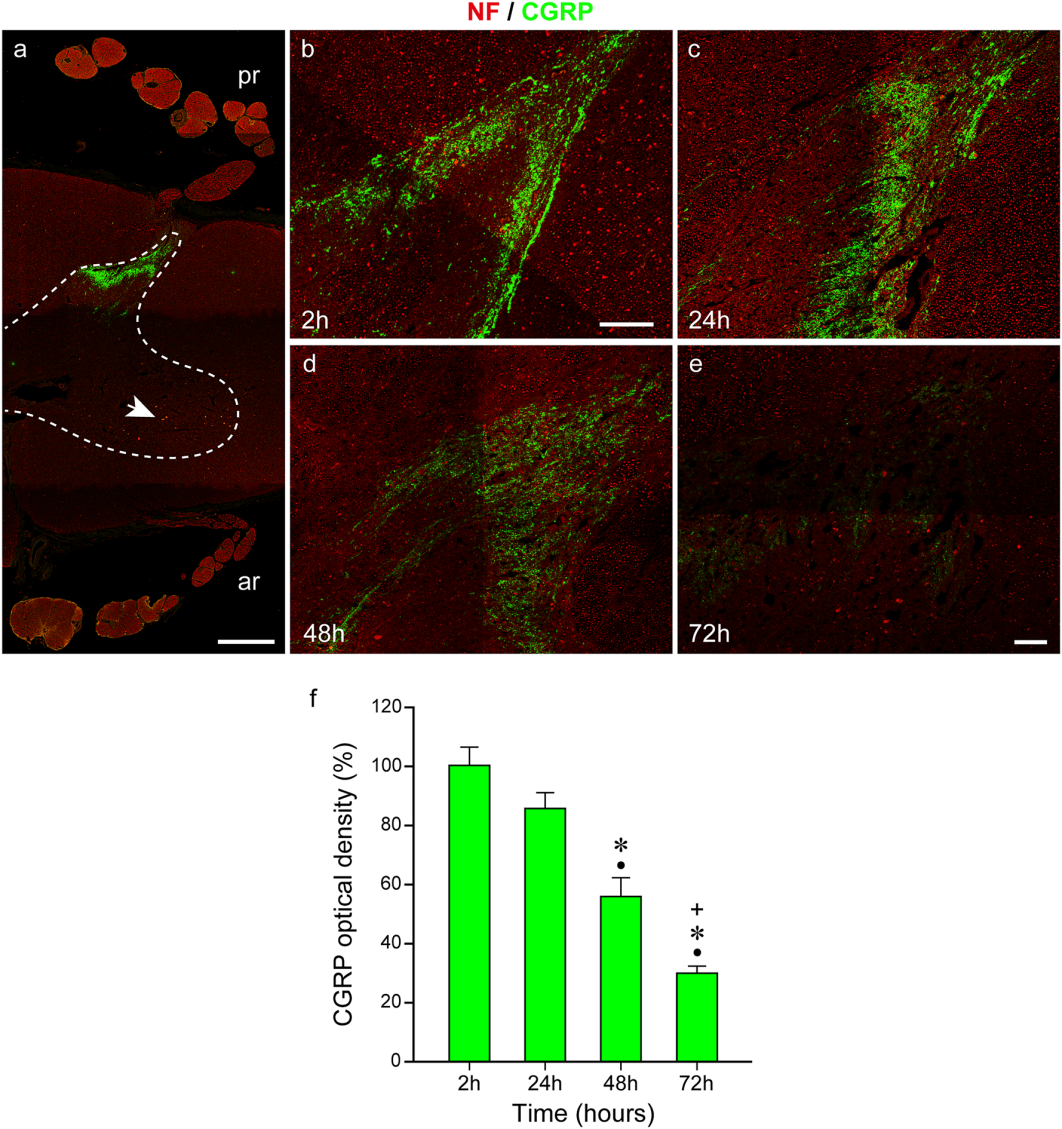
Double labeling with anti-neurofilament (NF) and anti-CGRP. **a** A segment of the human spinal cord at 24-h *postmortem* interval. Intense CGRP immunoreactivity is seen in the superficial layer of the posterior horn. Thedashed line demarcates the gray and white matter of the spinal cord. Anterior roots (ar), posterior roots (pr). Motoneuron cell bodies exhibiting lipofuscin autofluorescence are indicated by an* arrow*. **b–e** The CGRP-immunoreactivity in the dorsal horn of the organ donor (**b**) and body donors (**c–e**) at different *postmortem* intervals. At time points 2 h (**b**) and 24 h (**c**), a high level of CGRP is seen in the superficial layer of the dorsal horn. There is a downward trend in the staining intensity at 48 h (**d**) and at 72 h (**e**). Several tissue gaps are present 72 h *postmortem* (**e**).* Scale bars*: 1000 µm in **a**, 100 µm in **b**, and 100 µm in **e** for **c**, **d,** and **e**. **f** Bar chart showing the reduction of the CGRP signal over the *postmortem* interval. At time point 24 h, the CGRP intensity is 85% of the 2-h value (**f**, second bar). At time point 48 h, the CGRP signal intensity is 55% (**f** third bar) and at 72 h at about 30% (**f**, third bar) of the initial value (**f,** first bar).Black dots (●) indicate significant differences with time point 2 h,asterisks (*) indicate significant differences with time point 24 h, andplus signs (+) mark significant differences with time point 48 h. (one-way ANOVA test followed by Holm–Sidak post hoc method, *P* < 0.05)

After labeling the spinal cords of the organ donor and body donors with anti-neurofilament/anti-CGRP, we observed CGRP expression in the superficial layer of the posterior horn with decreasing CGRP signals in deeper layers. A panoramic image showing high CGRP expression in the posterior horn 24 h *postmortem* is presented in Fig. 8a. These findings are in line with data obtained from other organ donor spinal cords (Tschopp et al. 1984; Shiers et al. 2021). Quantitative analyses showed that in the 24-h body donor spinal cord, the CGRP brightness was slightly decreased and the value was 85% of the reference value measured in the 2-h organ donor spinal cord (Fig. 8a, b, c). CGRP intensity was only half of the 2-h value after 48 h and about 30% after 72 h (Fig. 8d, e, f). Structurally, large gaps were present in the posterior horn 72 h *postmortem* (Fig. 8e).

**Fig. 9:**
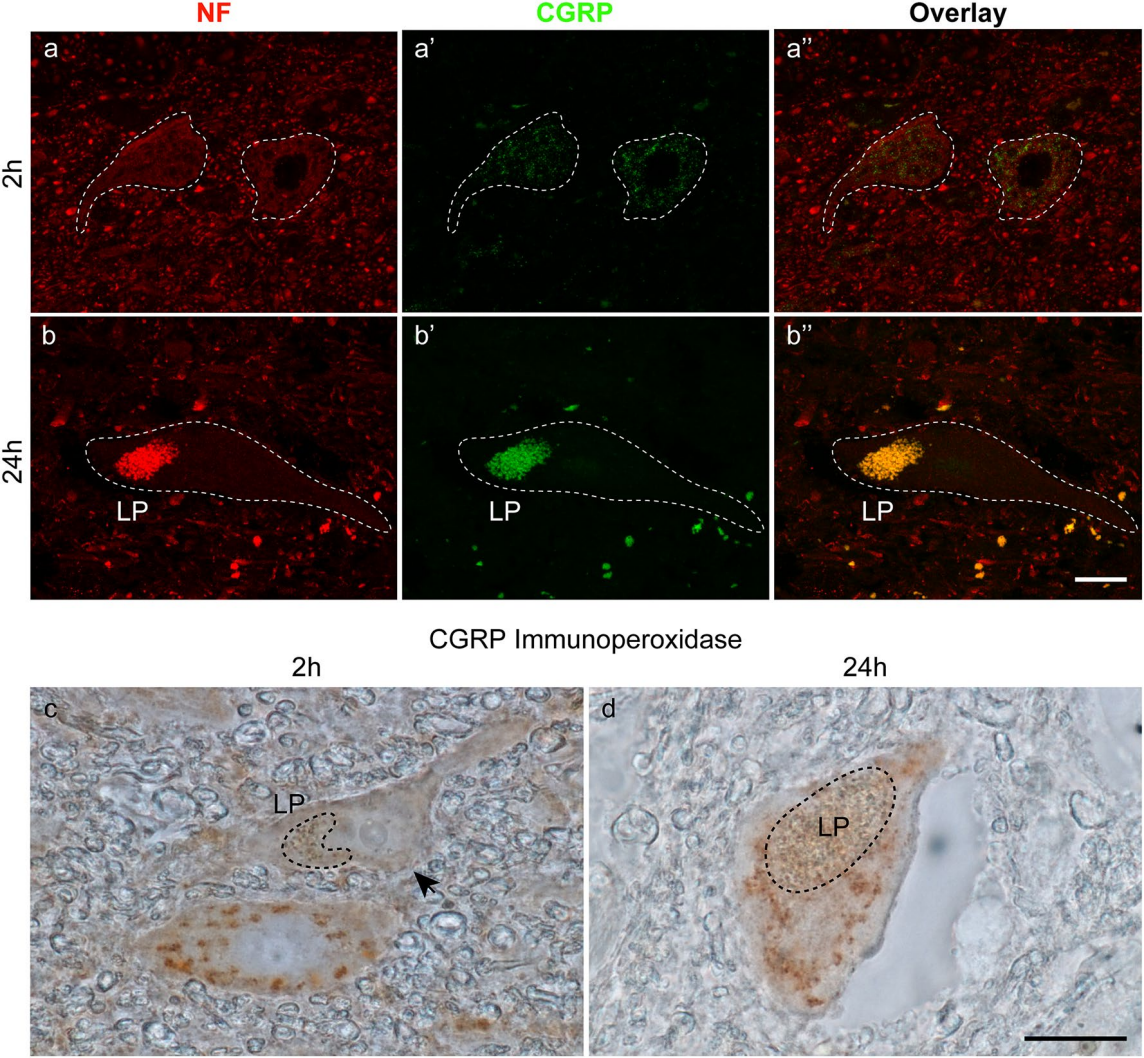
Double fluorescent labeling with anti-neurofilament (NF) and anti-CGRP and HRP immunohistochemistry with anti-CGRP. **a–a’’**, **b–b’’** Immunofluorescence of motoneurons stained with anti-neurofilament and anti-CGRP 2 h (**a–a’’**) and 24 h *postmortem* (**b–b’’**).Dashed lines demarcate motoneuron cell bodies. At time point 2 h (**a’**), motoneurons exhibit weak CGRP signals but CGRP signals are not visible 24 h *postmortem* (**b’**). Lipofuscin (LP) exhibiting red and green autofluorescence fills a part of the cell body (**b**, **b’**). Overlays of the neurofilament (**a**, **b**) and CGRP (**a’**, **b’**) staining are presented in **a’’**, **b’’**. The overlay shows lipofuscin autofluorescence in orange color due to the mix of the strong red and green autofluorescence signal (**b”**).Scale bar: 20 µm in **b’’** for **a–a’’** and **b–b’’**. **c**, **d** HRP immunohistochemistry with anti-CGRP showing motoneuron cell bodies 2 h (**c**) and 24 h *postmortem* (**d**)*.* Clod-like CGRP signals appear inbrown color and are visible at both *postmortem* intervals in cell bodies. In **c**, a cell body without a CGRP signal (arrow) is present. Lipofuscin (LP).Scale bar: 20 µm in **d** for **c** and **d**

In the spinal cord of the organ donor, some motoneuron cell bodies exhibited a punctate pattern of CGRP immunoreactivity (Fig. 9a–a’’) which, however, was much lower than the CGRP reactivity in the superficial layer of the posterior horn. The signal was only seen in the green fluorescence but not in the red fluorescence channel, thereby excluding lipofuscin autofluorescence which would be present in both channels. We observed that other motoneurons lacked CGRP signals (arrow in Fig. 9c). These findings are analogous to studies in the rodent spinal cord where it has been shown that a limited number of motoneurons express CGRP and the expression varies with age (Caldero et al. 1992; Blasco et al. 2020). No CGRP signals were detected in the motoneurons of the body donor spinal cords by immunofluorescence (Fig. 9b–b’’). Instead, in many motoneuron cell bodies, we observed lipofuscin autofluorescence in the green and red fluorescence channels (Fig. 9b–b’’). We tested HRP immunohistochemistry and by this technique, CGRP signals were observed in some motoneurons 24 h *postmortem* (Fig. 9d).

We next tested anti-neurofilament and anti-NeuN staining in the spinal cord. NeuN is a neuron-specific nuclear protein (Gusel'nikova and Korzhevskiy 2015). Hoechst 33342, a fluorescent dye that binds to the DNA, was used to counterstain cell nuclei. This approach allowed us to distinguish between neuronal cells expressing NeuN and Hoechst 33342 and non-neuronal cells expressing Hoechst 33342 alone. Because we observed a drop-down in the signal intensity over prolonged *postmortem* intervals in the staining combinations anti-neurofilament with (1) anti-ChAT, (2) anti-synaptophysin, and (3) anti-CGRP, the neurofilament/anti-NeuN staining experiment was exclusively done in the 2-h spinal cord of the organ donor and the 24-h spinal cord of the body donors.

**Fig. 10:**
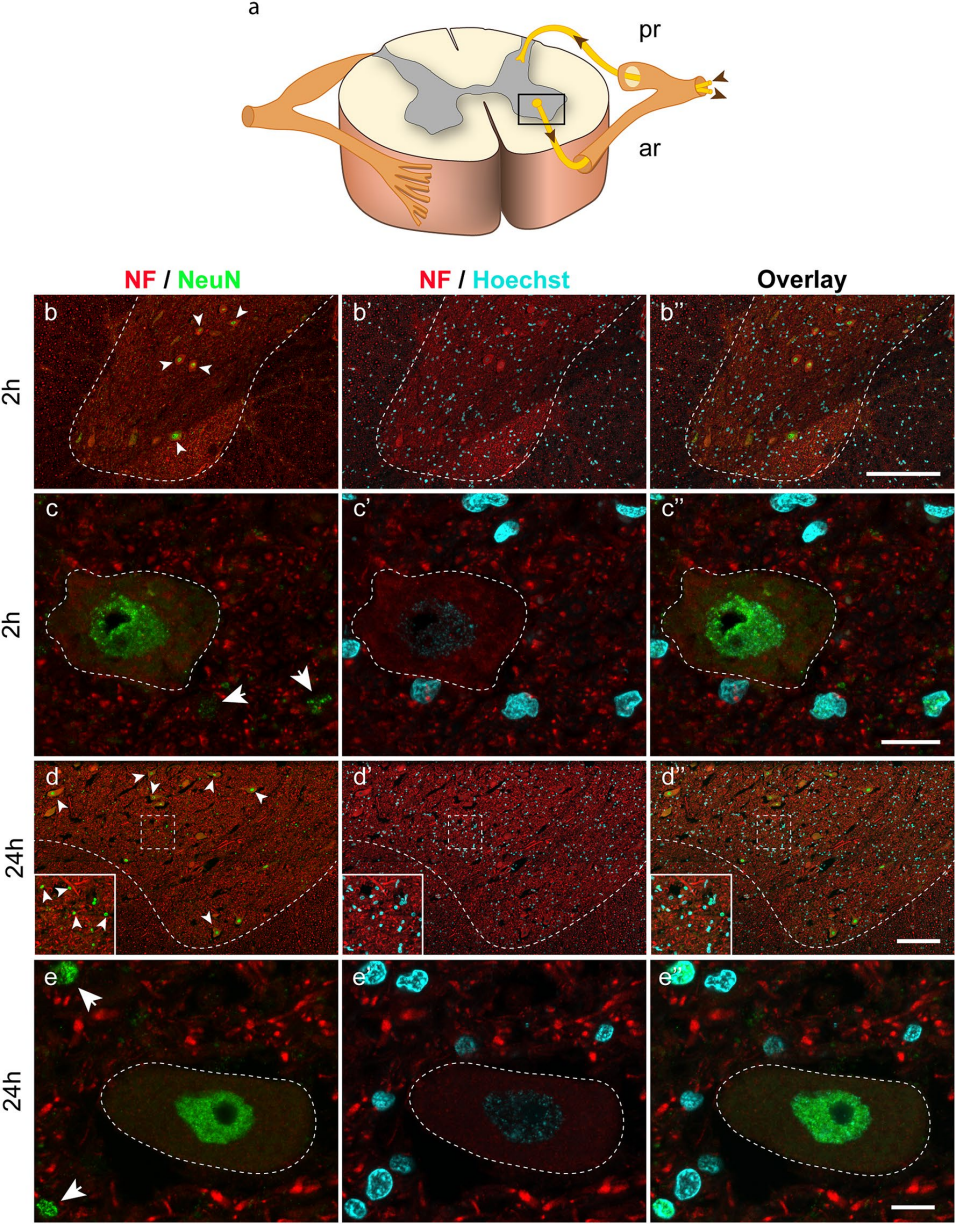
Triple labeling with anti-neurofilament (NF), anti-NeuN, and Hoechst 33342. **a** Schematic drawing with an inset illustrating the part of the spinal cord shown in figures **b–b’’**, and **d–d’’**. Posterior roots (pr), anterior roots (ar). **b–b’’**, **d–d’’** Overview images of the anterior horn 2 h (**b–b’’**) and 24 h *postmortem* (**d–d’’**) are shown in neurofilament and NeuN staining (**b, d**), neurofilament and Hoechst staining (**b’**, **d’**), and overlays (**b’’**, **d’’**). The gray and white matter are separated by adashed line. Motoneuron nuclei of the anterior horn express NeuN immunoreactivity (arrowheads in **b**, **d**). The inset in **d** shows small nuclei positive for NeuN (arrowheads). **c–c’’**, **e–e’’** High magnification images of motoneurons cell bodies (surrounded by adashed line) 2 h (**d–d’’**) and 24 h *postmortem* (**e–e’’**) in neurofilament and NeuN staining (**c**, **e**), neurofilament and Hoechst staining (**c’, e’**) and overlay (**c’’, e’’**). The motoneuron nucleus expresses NeuN (**c**, **e**) and is visualized by Hoechst stain in **c’** and **e’**. The overlays of **c** and **c’** and **e** and **e’** show co-localization of NeuN and the Hoechst signal. Small nuclei expressing NeuN (arrowheads in **c**, **e**) and Hoechst signals (**c’**, **e’**) are also visible.Scale bar: 200 µm in **b’’** for **b–b’’**; 15 µm in **c’’** for **c–c’’**; 200 µm in **d’’** for **d–d’’**; 15 µm in **e’** for **e–e’’**

Following fluorescent labeling, we visualized motoneurons in the anterior horn of the spinal cord*.* The inset in Fig. 10a indicates the region visualized in the spinal cord. In the organ donor and body donor spinal cord, the nucleus of the motoneuron cell body exhibited strong NeuN immunoreactivity (Fig. 10b–b”, c–c”, d–d”, e–e”). The NeuN signal in motoneurons co-localized with the Hoechst 33342 nuclear stain thereby confirming that the NeuN antibody is neuron-specific and nuclear-localized (Fig. 10c–c”, e–e”). We did not observe a difference in the anti-NeuN signal intensity 2 and 24 h *postmortem.* In addition to NeuN/Hoechst 33342-positive motoneurons, we found small nuclei which expressed NeuN/Hoechst 33342 as well (arrows in Fig. 10c, e). Interestingly, the NeuN signal was weaker in the organ donor spinal cord. We assume that these cells are interneurons, which for identification would require further neuronal markers.

### Triple-immunofluorescence

We tested the feasibility of triple immunofluorescence in the body donor spinal cord 24 h *postmortem*. Triple immunofluorescence included labeling with anti-neurofilament in combination **(**1) anti-synaptophysin/anti-CGRP, (2) anti-synaptophysin/anti-VAChT, and (3) anti-MBP/anti-CGRP. Anti-MBP visualizes the myelin sheath of axons and anti-VAChT cholinergic synapses.

**Fig. 11:**
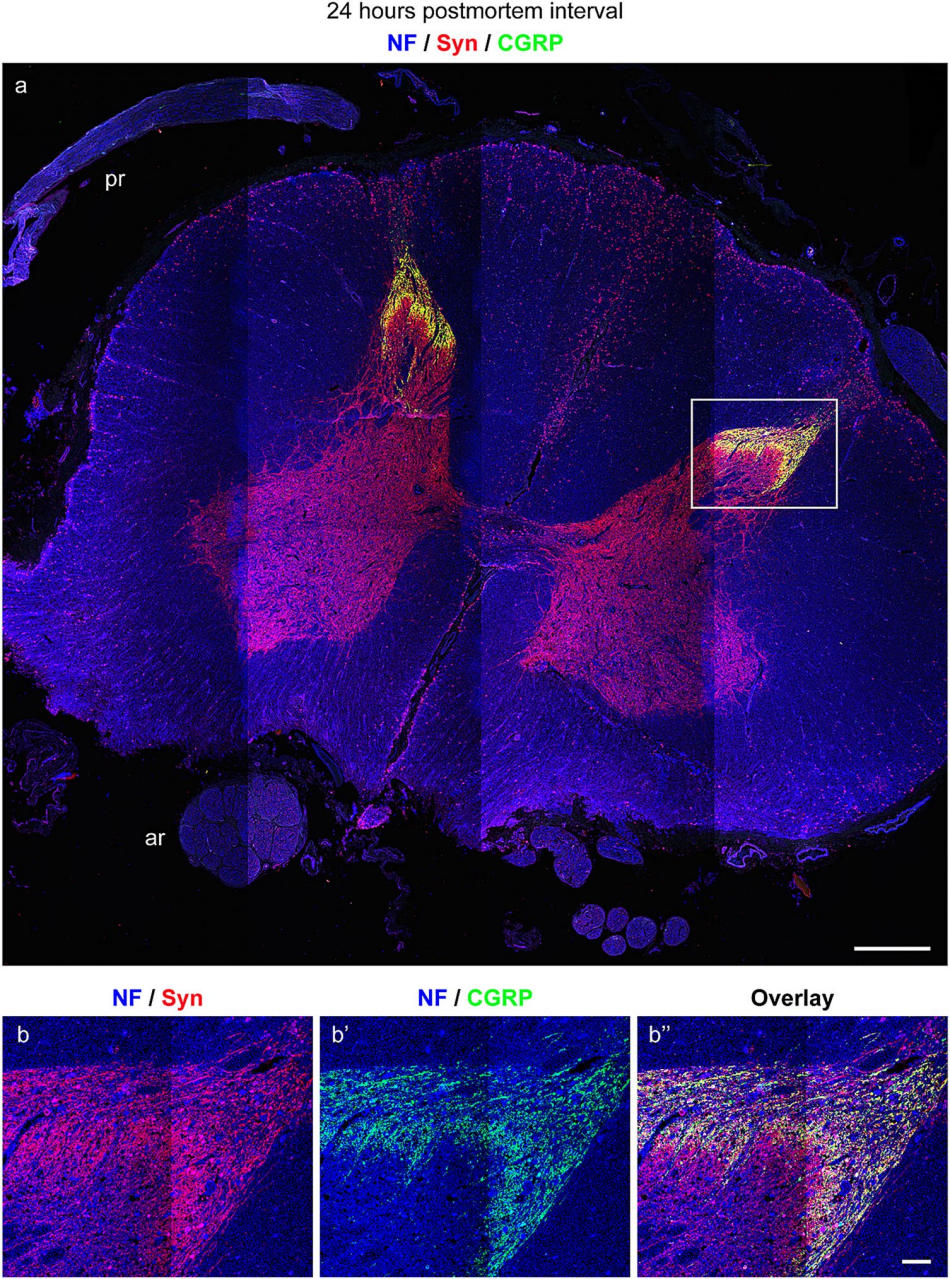
Triple labeling of a body donor spinal cord 24 h *postmortem* with anti-neurofilament (NF), anti-synaptophysin (syn), and anti-CGRP. **a** Cross section of a body donor spinal cord at 24 h *postmortem* interval. The whole gray matter exhibits synaptophysin immunoreactivity whereas CGRP is expressed in the superficial layer of the posterior horn. Anterior roots (ar), posterior roots (pr). **b–b’’** High-resolution images of the posterior horn (square box in **a**) in neurofilament/synaptophysin staining (**b**) neurofilament/CGRP staining (**b’**) and overlay **b’’**. The colocalization of synaptophysin (red) and CGRP (green) results in yellow color (**b’’**).Scale bars: 1000 µm in **a**, 100 µm in **b’’** for **b–b’’**

Following immunolabeling with anti-neurofilament/anti-synaptophysin/anti-CGRP, we observed co-localization of CGRP and synaptophysin in the superficial layer of the posterior horn of the gray matter (Fig. 11a, b–b’’).

**Fig. 12:**
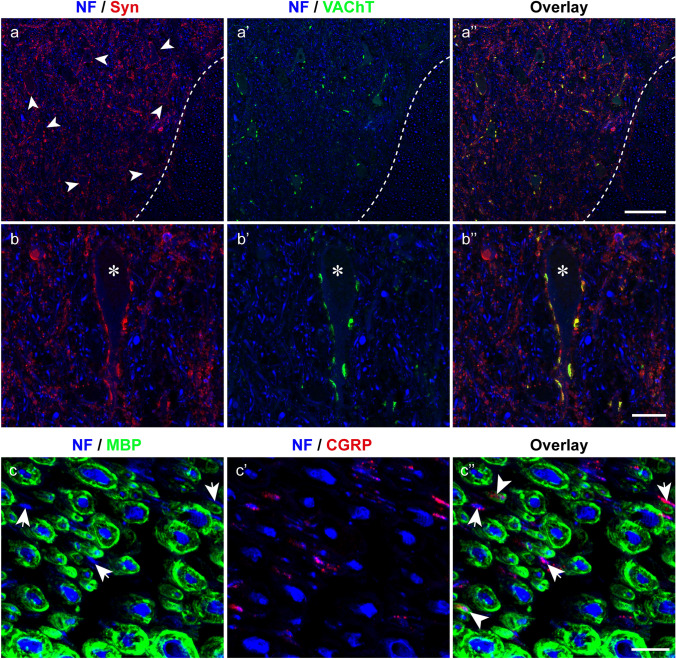
Triple labeling of a body donor spinal cord 24 h *postmortem*. (**a–a’’**, **b–b’’**) Labeling with anti-neurofilament (NF), anti-synaptophysin (Syn), and anti-VAChT. Cross section of the anterior horn (**a–a’’**) showing motoneurons (arrows in **a**) covered with synaptophysin (**a**) and VAChT (**a’**) positive synapses. The dashed line demarcates the border between the white and gray matter. The overlay (**a’’**) shows the mix of neurofilament, synaptophysin, and VAChT staining. (**b–b’’**) High-resolution image of a motoneuron cell body (*) covered with synapses expressing synaptophysin (**b**) and VAChT (**b’**). The overlay (**b’’**) shows colocalization of synaptophysin/VAChT in some synapses which appear in yellow color. (**c–c’’**) Labeling of the posterior root with anti-neurofilament, anti-MBP, and anti-CGRP. Most axons are covered with a myelin sheath (**c**) and only a few axons (arrows in **c**) are without myelin. Some thin axons express CGRP (**c’**) and are with myelin (arrowheads in **c’’**) or without a myelin sheath (arrows in **c’’**). Scale bars: 100 µm in **a’’** for **a–a’’**, 25 µm in **b’’** for **b–b’’**, 10 µm in **c’’** for **c–c’’**

In the staining combination anti-neurofilament/anti-synaptophysin/anti-VAChT, we identified VAChT-positive synapses on the cell bodies and proximal dendrites of spinal cord motoneurons. These VAChT-positive synapses resembled cholinergic C-boutons which are associated with spinal cord motoneurons in many mammalian species (Nagao et al. 2003; Witts et al. 2014). VAChT positive synapses expressed synaptophysin as well. Other synaptophysin-positive synapses on anterior horn motoneurons lacked VAChT immunoreactivity (Fig. 12a–a’’, b’–b’’).

After staining combination anti-neurofilament/anti-MBP/anti-CGRP, we visualized myelinated nerve by anti-MBP and putative nociceptor fibers by anti-CGRP in the posterior roots. We observed that most axons in the posterior root were covered by a myelin sheath (Fig. 12c) and few axons expressed CGRP (Fig. 12c’). All CGRP-positive axons were of small diameter, and they were with or without a myelin sheath (Fig. 12c’’).

**Fig. 13:**
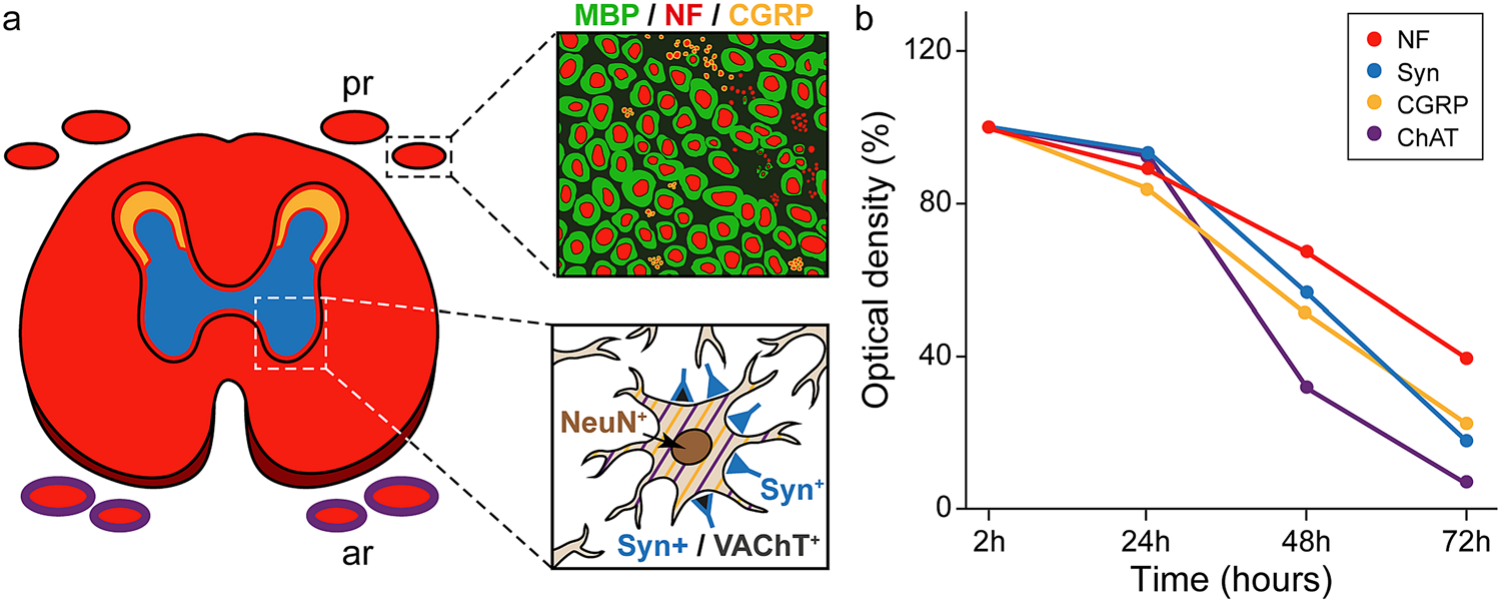
Schematic diagram summarizing the staining pattern (**a**) and decay (**b**). **a** Anti-neurofilament (red) is present in the white and grey matter (outlined in black color) of the spinal cord as well as in axons of the anterior (ar) and posterior roots (pr). Axons of the anterior roots express ChAT as well (red/purple) Synaptophysin (blue) is expressed throughout the gray matter and CGRP (yellow) in the superficial layer of the dorsal horn. Lower inset of **a** shows anterior horn motoneurons covered with synapses that express synaptophysin/VAChT (blue andblack) and other synapses expressing synaptophysin (blue) only. Motoneuron cell bodies express CGRP and ChAT (purple yellow lines) and the nucleus NeuN (brown). Upper inset of **a** showing a detail of the posterior root with myelinated axons (red/green) and unmyelinated axons (red) some of which express CGRP (red/yellow). (**b**) Histogram showing the mean decay of the staining intensity in different antibodies over *postmortem* intervals

Taken together, by multicolor immunolabeling with a series of neuronal marker (antibodies against neurofilament, ChAT, synaptophysin, CGRP, NeuN, VAChT, and MBP), we have visualized different neuronal elements in fresh spinal cord from organ donor and body donors at different *postmortem* intervals and demonstrated that the image quality is almost equal in the spinal cord from organ donor and body donors 24 h *postmortem*. By HRP immunohistochemistry, the absence of ChAT and CGRP fluorescence signals in anterior horn motoneurons 24 h *postmortem* could be compensated. With prolonged *postmortem* interval (48 and 72 h), the signal intensity of neuronal markers diminished but this downward trend was antibody-specific and moderate in the neurofilament antibody and strong in the ChAT antibody whereas values for CGRP and synaptophysin antibodies were in between. Additionally, we showed that automated quantification of axons is possible with high accuracy 24 h after death. The results of the present study are summarized in Fig. 13.

At this point, it is also important to note that in our immunofluorescence assays, only minimal staining variability was observed between individuals of the same *postmortem* group except for one case in the 72-h group where stronger immunofluorescence was observed than in other cases of this group.

## Discussion

Immunohistochemistry is a powerful tool in analyzing human neuronal tissue at the molecular level. Typically, neuronal tissue is harvested from organ donors (Tschopp et al. 1984; Eftekhari and Edvinsson 2011; Gesslbauer et al. 2017; Mioton et al. 2019; Stakenborg et al. 2020; Dominguez-Alvaro et al. 2021; Shiers et al. 2021) or body donors (Rein et al. 2013, 2015, 2020; Pascual-Font et al. 2016; Lienbacher et al. 2019). Harvesting neuronal tissue from organ donors requires a high administrative workload and is often prohibited. Contrary, tissue from body donors is more easily available and can be an alternative source for research.

The present study aimed to define a *postmortem* time window during which accurate immunohistochemical analyses are possible in the spinal cord of body donors. Here we demonstrated by immunofluorescence/HRP immunohistochemistry that precise identification of multiple, molecularly different neuronal elements was possible in the spinal cord of body donors until 24 h *postmortem,* and generated image data were qualitatively almost equal to those obtained from the organ donor spinal cord with 2-h *postmortem* interval. Although present data are related to tested molecules, our findings provide a useful guide for immunolabeling assays in neuronal tissue from organ donors.

Today there is increasing interest in analyzing human neuronal tissue including the brain (Waldvogel et al. 2006; Blair et al. 2016), spinal cord (Tschopp et al. 1984; Eftekhari and Edvinsson 2011; Shiers et al. 2021), and peripheral nerves (Gesslbauer et al. 2017; Mioton et al. 2019; Stakenborg et al. 2020) by immunohistochemistry to better understand neuronal function. As there is a need for high tissue quality, fresh tissue from organ donors is often preferred, although tissue collection is very challenging. In the present study, we showed that robust neurochemical information can be obtained from body donor spinal cords 24 h *postmortem*. By multi-color immunofluorescence, up to three molecules could be visualized 24 h after death in the same tissue sample and this offered the opportunity to detect colocalization of molecules and to perform counts of multiple neuronal elements. The high accuracy of the neurochemical information 24 h *postmortem* was confirmed by automated quantification. Specifically, 99.5 out of 100% of the axons leaving the spinal cord via the anterior root exhibited ChAT immunoreactivity and the same value (more than 99%) of cholinergic axons was counted in fresh spinal cord obtained from heart-beating organ donors (Gesslbauer et al. 2017). This important information suggests that neuronal tissue from body donors is suitable for qualitative and quantitative analyses of axonal components in mixed peripheral nerves and could substitute tissue from organ donor tissue, which was used recently for such analyses (Gesslbauer et al. 2017; Mioton et al. 2019; Stakenborg et al. 2020). Altogether, the present findings underline that accurate interpretation of immunohistochemical data is possible in neuronal tissue harvested from body donors.

It has been shown in rodents that the cell bodies of the anterior horn motoneurons express ChAT (Friese et al. 2009) and at least a subpopulation of them express CGRP (Caldero et al. 1992; Blasco et al. 2020). By immunofluorescence, no ChAT and CGRP signals were detected in the motoneuron cell bodies of the 24-h body donor spinal cord whereas weak ChAT and CGRP immunoreactivity was detected in the organ donor. By HRP immunohistochemistry, we visualized ChAT and CGRP in some but not all motoneuron cell bodies 24 h *postmortem.* These findings indicate that ChAT and CGRP, although at a low level, were still present 24 h *postmortem* in motoneuron cell bodies and it is recommended to consider HRP immunohistochemistry when performing *postmortem* studies.

As expected, immunofluorescence signals decreased over *postmortem* intervals, yet the staining intensity in the 48-h spinal cord was acceptable and high enough for qualitative information. Similar *postmortem* changes were observed in organ donor brains and reduced but stable immunoreactivity was present in neurons visualized with anti-tubulin and anti-neurofilament 48 h *postmortem* (Blair et al. 2016). It is however important to note, that the signal reduction observed here was antibody-specific and strongest in the anti-ChAT antibody. This signal reduction had consequences when performing quantitative analyses. Specifically, 48 h *postmortem* only 90 out of 100% of cholinergic axons in the anterior root were identified, indicating that in the missing axons, ChAT signals were at a level too weak for being detected by the analysis software. These findings along different *postmortem* intervals indicate that qualitative immunohistochemical information can be obtained from body donor neuronal tissue over a period longer than 24 h after death but there are limitations regarding quantitative analyses.

Based on our observations, a *postmortem* interval of 72 h is too long for proper immunohistochemistry in neuronal tissue from body donors. At this time point, spinal cords exhibited substantial morphological changes due to autolysis and with the exception of the neurofilament antibody, immunohistochemical signals in the other antibodies (anti-ChAT, anti-synaptophysin, and anti-CGRP) were very low i.e., between 20 and 40% of the initial value. These *postmortem* changes are consistent with findings in the brain, where the synaptophysin signal significantly decreased 72 h after death (Liu and Brun 1995), and do not support data in autonomic nerve fibers of the heart where the signal drop was observed not until the 7th day *postmortem* (Chow et al. 1998). The low tissue quality coupled with strong immunohistochemical signal loss questions the scientific value of information obtained from human neuronal tissue 72 h after death.

The antibody-specific signal reduction observed in the present study is consistent with two older publications evaluating the *postmortem* effects on the immunohistochemistry of autonomic nerve fibers in humans (Gu et al. 1985; Chow et al. 1998) and a recent study evaluating this effect in the brain (Waldvogel et al. 2006; Blair et al. 2016). After death, tissue proteins degrade and this reduces the binding capacity of antibodies and consequently the signal strength (Ramos-Vara and Miller 2014). Because the degradation of tissue proteins is temperature dependent, it is important to refrigerate body donors after arrival at body donor facilities. The stability of different tissue proteins varies in *postmortem* tissue (Waldvogel et al. 2006) and there are indications that structural proteins like neurofilament (Karlsson et al. 1991) and cell membrane proteins like synaptophysin (Wiedenmann and Franke 1985) are better preserved than soluble enzymes (ChAT) (Bruce and Hersh 1987). This was confirmed in the present study because the signal loss was less dramatic in neurofilament and synaptophysin staining compared to ChAT staining. CGRP a neuromodulator protein is similar well preserved as synaptophysin as shown here.

An additional discovery of the present study was that the signal intensity of the ChAT antibody varied in different compartments of the motoneuron. Specifically, in the 24-h spinal cord high levels of ChAT were present in the axons of the anterior root and in the synapses of the motoneuron cell bodies whereas low or no ChAT signal was inside the motoneuron cell bodies. This spatial variation of the staining intensity suggests that the ChAT enzyme disintegrates *postmortem* with variable speed in different motoneuron compartments and based on the low antibody signal, this process is quicker in cell bodies than in axons and synapses. Taken together, information on the stability of different neuronal proteins and variations of the protein stability in neuronal compartments is crucial and must be considered when evaluating immunohistochemistry in human *postmortem* tissue.

## Conclusions

Here, we have demonstrated that multi-color immunofluorescence coupled with HRP immunohistochemistry allows detailed qualitative and quantitative analyses in the spinal cord from body donors 24 h *postmortem* and at least qualitative analyses are possible until 48 h *postmortem*. These data underline that neuronal tissue from body donors can be an alternative source for immunohistochemical analyses in basic and clinical research. Moreover, qualitative and quantitative immunohistochemical information obtained from normal neuronal tissue of body donor can be compared with immunohistochemical information from cases with neuronal disorders.

## Supplementary Information

Below is the link to the electronic supplementary material.

Supplementary file1 The lipofuscin autofluorescence signal depends on the setting in the CLSM. (**a**–**a’’**, **b**–**b’’**) Showing identical images of motoneuron cell bodies (demarcated with dashed lines) labelled with anti-neurofilament (NF, **a**, **b**) and anti-synaptophysin (Syn; **a’**, **b’**), and in overlays (**a’’**, **a’’**). In **a** and **b**, the red lipofuscin autofluorescence is visible and the strength is the same in **a** and **b** because CLSM laser settings were identical. In a’ and b’ the green lipofuscin autofluorescence is visible. In b the synaptophysin signal is oversaturated. In b’ the laser intensity was reduced resulting in lower green lipofuscin autofluorescence and sharp synaptophysin signals. Overlays (**a’’**, **b’’**) show that the mixture of strong red and green autofluorescence (**a’’**) results in orange color whereas strong red but low green autofluorescence (**b”**) results in red color. Scale bars: 20 μm in **b’’** for **a**–**a’**, **b**–**b’’** (TIF 10801 KB)
