# Supplementary figures and images for: Spinal cord from body donors is suitable for multicolor immunofluorescence
Source: Histochem Cell Biol. 2022 Oct 6;159(1):23–45. doi: 10.1007/s00418-022-02154-5 (PMC9899749; doi:10.1007/s00418-022-02154-5)

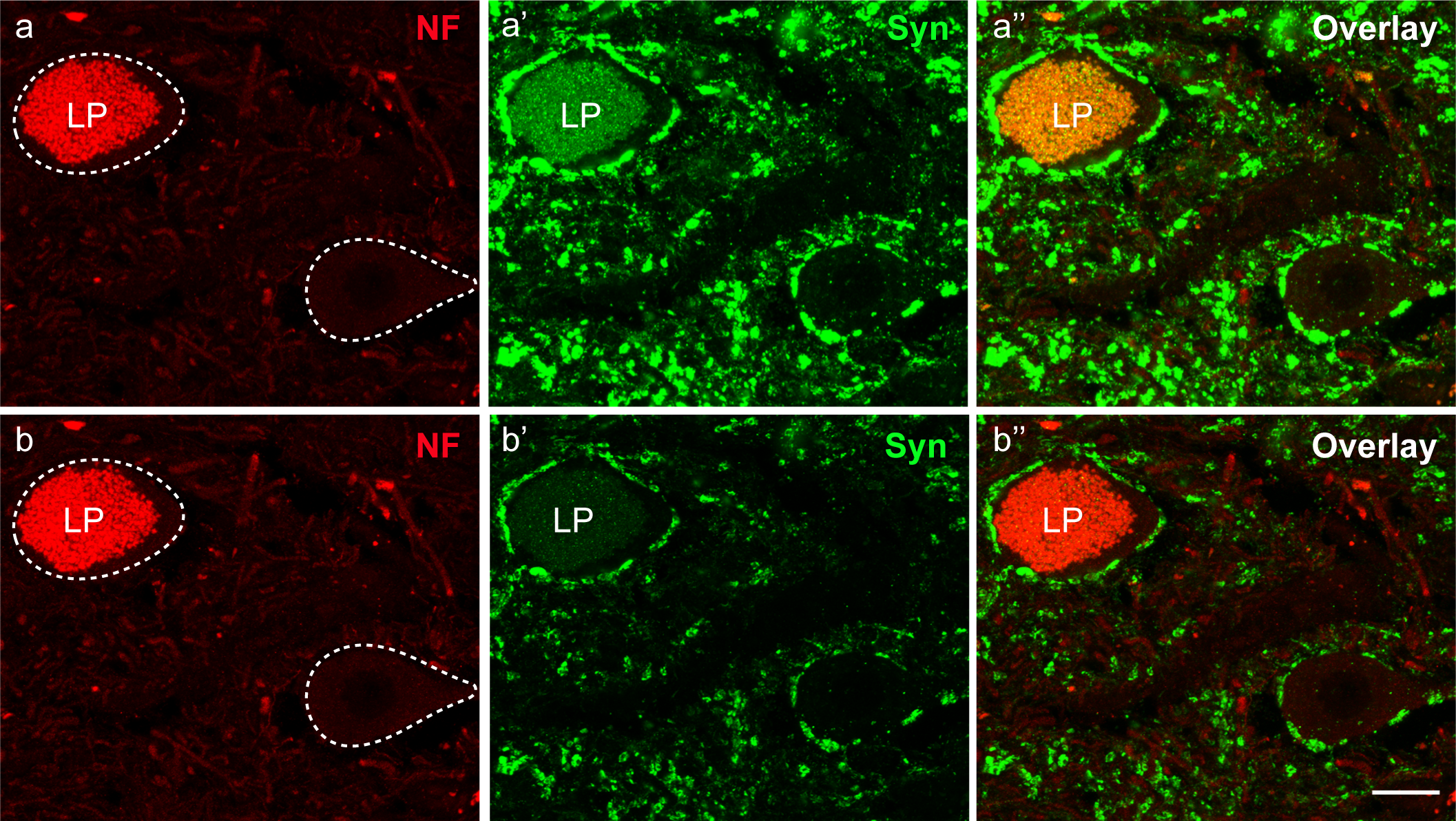

Supplement: Supplementary file 1 — Supplementary file1 The lipofuscin autofluorescence signal depends on the setting in the CLSM. (a–a’’, b–b’’) Showing identical images of motoneuron cell bodies (demarcated with dashed lines) labelled with anti-neurofilament (NF, a, b) and anti-synaptophysin (Syn; a’, b’), and in overlays (a’’, a’’). In a and b, the red lipofuscin autofluorescence is visible and the strength is the same in a and b because CLSM laser settings were identical. In a’ and b’ the green lipofuscin autofluorescence is visible. In b the synaptophysin signal is oversaturated. In b’ the laser intensity was reduced resulting in lower green lipofuscin autofluorescence and sharp synaptophysin signals. Overlays (a’’, b’’) show that the mixture of strong red and green autofluorescence (a’’) results in orange color whereas strong red but low green autofluorescence (b”) results in red color. Scale bars: 20 μm in b’’ for a–a’, b–b’’ (TIF 10801 KB) [file 418_2022_2154_MOESM1_ESM.tif]
